# The colored Hanbury Brown–Twiss effect

**DOI:** 10.1038/srep37980

**Published:** 2016-12-06

**Authors:** B. Silva, C. Sánchez Muñoz, D. Ballarini, A. González-Tudela, M. de Giorgi, G. Gigli, K. West, L. Pfeiffer, E. del Valle, D. Sanvitto, F. P. Laussy

**Affiliations:** 1 CNR NANOTEC–Institute of Nanotechnology, Via Monteroni, 73100 Lecce, Italy; 2 Departamento de Física Teórica de la Materia Condensada and Condensed Matter Physics Center (IFIMAC), Universidad Autónoma de Madrid, 28049 Madrid, Spain; 3 Max–Planck Institut für Quantenoptik, 85748 Garching, Germany; 4 Department of Electrical Engineering, Princeton University, Princeton, New Jersey 08544, USA; 5 Russian Quantum Center, Novaya 100, 143025 Skolkovo, Moscow Region, Russia

## Abstract

The Hanbury Brown–Twiss effect is one of the celebrated phenomenologies of modern physics that accommodates equally well classical (interferences of waves) and quantum (correlations between indistinguishable particles) interpretations. The effect was discovered in the late thirties with a basic observation of Hanbury Brown that radio-pulses from two distinct antennas generate signals on the oscilloscope that wiggle similarly to the naked eye. When Hanbury Brown and his mathematician colleague Twiss took the obvious step to propose bringing the effect in the optical range, they met with considerable opposition as single-photon interferences were deemed impossible. The Hanbury Brown–Twiss effect is nowadays universally accepted and, being so fundamental, embodies many subtleties of our understanding of the wave/particle dual nature of light. Thanks to a novel experimental technique, we report here a generalized version of the Hanbury Brown–Twiss effect to include the frequency of the detected light, or, from the particle point of view, the energy of the detected photons. Our source of light is a polariton condensate, that allows high-resolution filtering of a spectrally broad source with a high degree of coherence. In addition to the known tendencies of indistinguishable photons to arrive together on the detector, we find that photons of different colors present the opposite characteristic of avoiding each others. We postulate that fermions can be similarly brought to exhibit positive (boson-like) correlations by frequency filtering.

The science of photon correlations—quantum optics—started with the theory that Glauber developed to account for the conclusive observation by Hanbury Brown and Twiss^1^ that photons from thermal light detected at the single particle level do indeed exhibit bunching in their arrival time, in the same way as radio-waves correlated in intensities^2^. The word “coherent” then changed from the meaning as used by HBT^3^ (to mean monochromatic) to that of Glauber^4^ (to mean of uncorrelated photons). The fact that initially unrelated photons, emitted maybe from different stars in different galaxies, would exhibit a bunching effect, that is, a tendency of arriving together on the detector, provoked much outrage and incredulity in many of the prominent physicists of the time^5^, despite having an immediate classical interpretation in terms of constructive interferences^6^. This phenomenon was quickly understood by Purcell^7^ as, not only compatible with the particle point of view, but also required by it, being associated to the positive pair-correlation between bosons caused by their indistinguishability. A complete formalization of the underlying principle has been Nobel-prize winning^8^, culminating with a now central quantity in quantum optics, the “Glauber’s second-order coherence function *g*^(2)^” defined as:


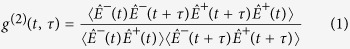


with 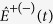 the negative (positive) frequency part of the Heisenberg electric field operator at time *t* and *τ* the time delay between detections (we omit position dependence for simplicity). This quantity describes the statistical distribution between photons in their stream of temporal detection. Other properties of the photons can be included, e.g., their position^1^ or polarisation^9^, with applications spanning from atomic interferometry^10^ to entangled photon pair generation^11^.

## Correlations when retaining the color of the photons

Of all the possible additional variables that one can include or retain when correlating the photons, one is so intertwined with the temporal information as to define a special case of its own: it is the energy of the photon (or, equivalently in the wave picture, its frequency). This is a characterisation of a different type than position or polarisation, since time and frequency are conjugate variables. Frequency–resolved correlations are furthermore observables that cannot be associated to a given quantum state, as they also bring information on the dynamics of emission. This results in a wider and unifying perspective of photon correlations. The formal theory of time and frequency resolved correlations, established in the 80s^12^,^13^,^14^,^15^, upgrades Eq. (1) to the two-photon frequency correlations:


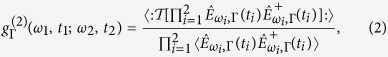


where


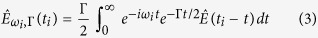


is the electric field after passing through a filter with frequency component *ω*_*i*_ and width Γ at time *t*_*i*_, and 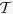, (resp.:) refers to time (resp. normal) ordering. Equation (2) provides the tendency of a correlated detection of one photon of frequency *ω*_1_ at time *t*_1_ with another photon of frequency *ω*_2_ at time *t*_2_. We consider here Lorentzian filters but this discussion applies to other types, such as square filters^16^. Frequency-resolved photon correlations are an increasingly popular experimental quantity, with already many measurements performed, although for fixed sets of frequencies, merely by inserting filters in the paths of a standard Hanbury Brown–Twiss setup^17^,^18^,^19^,^20^,^21^,^22^. This measurement reveals its conceptual importance, however, when spanning over all possible combinations of energies, giving rise to a so-called “two-photon correlation spectrum” (2PS)^23^,^24^. Considering the most common case of coincidences—*τ* = 0 in Eq. (1) and *t*_1_ = *t*_2_ in Eq. (2)—one turns in this way a single number, 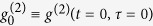, into a full landscape 
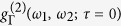
 of correlations. The quantity defined by such a landscape (the 2PS) acquires a fundamental meaning by revealing certain physical features^23^,^24^, in the same way that the normal spectrum is meaningful because its an observable that spans over a frequency range.

## Results

In this work, we report the complete HBT effect extended to the full frequency-frequency map. We find that—in addition to the original observation of positive correlations for identical photons reported by the fathers of the effect and now routinely reproduced in a multitude of quantum-optical platforms worldwide—photons also manifest anticorrelations when they have different energies. Our results are a direct extension of the original HBT effect, that is the particular case of the diagonal on our 2PS. At such, it bears similar attributes as well as counter-intuitive consequences. Namely, two photons detected from two different sources manifest anticorrelations if detected in different frequency windows (namely, on opposite sides of their mean energy) as compared to the unfiltered detection. If the sources are coherent, so that the unfiltered detection presents no correlation, the frequency-filtered photons exhibit anticorrelations: the detection of photons of a given color makes it less likely to detect photons of the other color. This behaviour is rooted in the bosonic fabric through what we will introduce as the “boson form factor”. Like the original HBT effect, our findings can be interpreted both from a quantum or a classical point of view, and being due to boson statistics, represent a fundamental backbone of every experiment involving frequency-resolved correlations. This result is therefore of deep relevance for any measurement of this kind, becoming of great interest in scenarios like the study of fluctuations^20^ or the harvesting and use of quantum correlations that only emerge in the frequency-frequency domain^21^,^22^,^23^,^25^.

**Figure 1:**
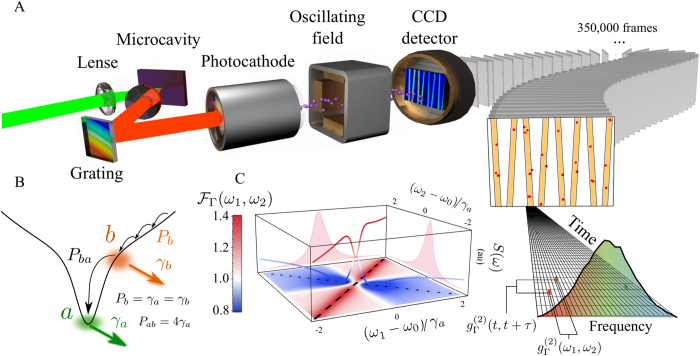
Principle and Setup of time- and frequency-resolved photon correlations. (**A**) Sketch of the experiment: the reflected light from a microcavity is dispersed onto a streak camera detecting at the single-photon level and stored in individual frames, whose post-processing allows to build photon-correlation landscapes. (**B**) Sketch of the theory: a laser excites non-resonantly the lower polariton dispersion, creating a reservoir of hot excitons *b* that condense into the ground state *a* at the minimum of the branch. The rate of exciton injection is given by *P*_*b*_. Excitons decay at a rate given by *γ*_*b*_, and polaritons at the bottom of the branch, at a rate *γ*_*a*_. The transfer rate from the excitons of the reservoir to the polaritons is given by *P*_*ba*_. See section IV of the Supplemental Material. (**C**) Boson form factor 
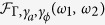
 (see Eq. 7), i.e., time-integrated 2PS for the spontaneous emission of a coherent state with *g*^(2)^ = 1, providing the backbone for the experiment. The diagonal (dashed line) and antidiagonal (dotted line) exhibit bunching and antibunching, respectively.

We have measured such anticorrelations between single photons emitted from a macroscopic out-of-equilibrium ensemble of exciton-polaritons pumped around the threshold of condensation. Polaritons are strongly-coupled light-matter bosonic particles in a semiconductor microcavity^26^. Such a source is more convenient than a laser because it is, for our purposes, essentially a laser with a broad linewidth, thereby allowing the spectral filtering. Besides, they have enjoyed thorough studies of their coherence properties, including at the quantum optical level^27^,^28^,^29^. At the low powers that we have used, the polariton condensate is free from their typical complications: interactions, multi-mode condensations, effect of the reservoir, etc. We are therefore confident that it behaves essentially as a laser and that any other coherent source would provide the same results. The experiment is based on a streak camera setup that detects individual photons from the spontaneous emission of an ensemble of polaritons maintained in a non-equilibrium steady state under continuous wave excitation. This is the first time that such a technique is used in the continuous pumping regime. The setup is sketched in Fig. 1A: light coming from the steady state of polaritons is dispersed by a spectrometer and is directed into the streak camera that is able to detect single photon events, as has already been demonstrated with standard photon correlations in time domain only and under pulsed excitation^30^. The sweeping in time and dispersion in energy allow the simultaneous recording of both the time and frequency of each detected photon in successive frames that are post-processed to calculate intensity correlations. The storage capacity on the streak camera can be increased eight-fold by adding a small horizontal drift in time allowing to collect the signal from the intersection of a sine-wave with each frame of the CCD detector. For large-amplitude vertical sweeps, only the linear part of the sine function is recorded as an oblique trace (nonlinear deviations could easily be corrected for). Figure 1A shows the 8 sweeps per frame as tilted orange stripes, with red dots indicating single photon events. Within each sweep, a time of 1536 ps is spanned in the vertical direction (3.2 ps per pixel), while the photon energy, obtained by coupling a spectrometer to the streak camera, is measured as horizontal pixel positions within each sweep (each sweep covers a total energy range of 456.7 µeV, with 10.6 µeV per pixel). With the time- and energy-range used in this experiment, the overall temporal and energy resolution of the setup are of 10 ps and 70 µeV, respectively. Correlation landscapes are obtained from coincidences between these clicks, with an average of ≈1.69 clicks per sweep in a total of 350 000 frames. All the analysis is done with the raw data only: there is no normalisation and the correlations go to 1 at long time self-consistently. A scheme of the emitter is shown in Fig. 1B: polaritons relax into the ground state from a reservoir of high energy polaritons injected by a continuous wave off-resonant laser. The constant losses through the cavity mirror allow to study the steady-state correlations. Both the principle of the measurement and the technique are general and should allow, with optimisation, to shed new light in already well known systems in quantum optics, starting with the interesting non-classical properties displayed by quantum sources. The time resolution is outstanding as it can be less than 1 ps in a time window of 100 ps per sweep. In the case of spectral diffusion of fluctuating systems^20^, for instance, this would improve temporal precision by two orders of magnitude.

**Figure 2:**
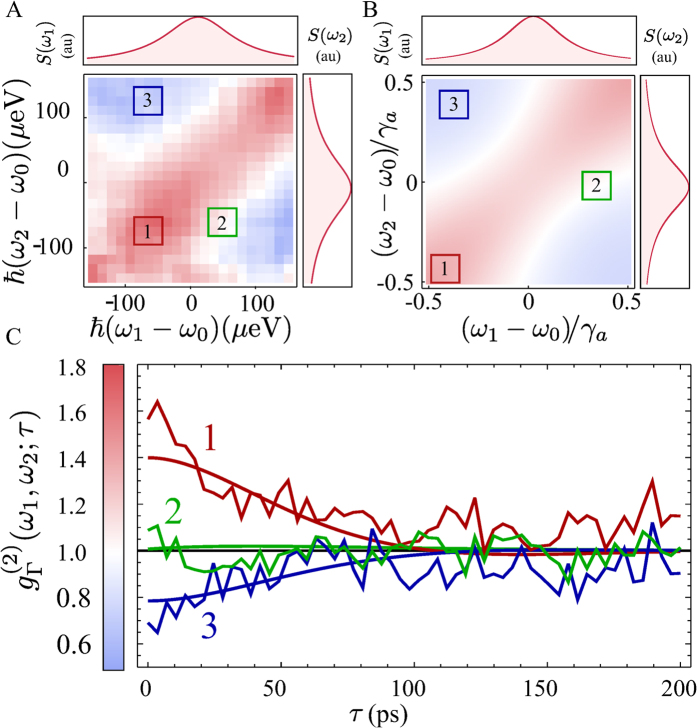
Two-photon correlations spectra. (**A**) Experimental observation of 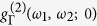 for the spontaneous emission from a steady-state of polaritons. (**B**) Theoretical calculation of 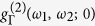 from the model of condensation of polaritons sketched in Fig. 1B, showing a remarkable agreement. (**C**) Time-resolved correlation for the three regions marked in the color map: (i) on the diagonal (*ω*_1_ = *ω*_2_) exhibiting bunching, (ii) in the region of transition with no correlation, (iii) correlating opposing elbows, exhibiting antibunching.

Figure 2A shows the experimental 2PS for the polariton state at *τ* = 0 together with the theoretical prediction, that is shown in Fig. 2B and was computed from the steady state emission of the model of condensation sketched in Fig. 1B using a master equation and the recently developed sensors method^31^ (see Section IV of the Supplemental Material). Figure 2C depicts the temporal correlations for three points of the (*ω*_1_, *ω*_2_)–space, both for the experiment and the theoretical model, demonstrating an outstanding time precision in the scale of picoseconds. A clear evolution of the correlations from bunching (
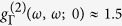
 in region 1) to antibunching (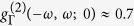 in region 3) is observed. In general, an excellent agreement with the theory is obtained, especially for the salient features which are diagonal bunching and antidiagonal antibunching.

## Discussion

**Figure 3:**
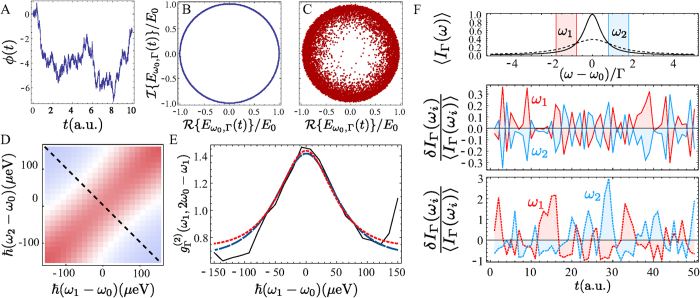
(**A**) The random-walk evolution of diffusing phase of a field 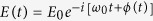, with 〈*e*^*i*(*ϕ*(*t*+*τ*)−*ϕ*(*t*))^〉 = *e*^−*γτ*^. (**B**) *E*(*t*) in phase space over different times. (**C**) Phase fluctuations are converted into intensity fluctuations after frequency filtering *E*(*t*). (**D**) Fitting of the experimental 2PS by equation (6), with fitting parameters *γ* ≈ 193 µeV, Γ ≈ 134 µeV. The colorscale is that of Fig. 2. (**E**) 2PS along the dashed line in (**D**) for the experiment (straight, black), the fitting for the phase diffusing field (long dashed, blue) given by Eq. (6) and the fitting of the form factor 
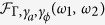
 (short-dashed, red). Despite not being an exact theoretical description for this experiment, the form factor agrees very well with the data for the parameters *γ* ≈ 99 µeV, *γ*_*ϕ*_ ≈ 440 µeV, Γ ≈ 17 µeV. (**F**) Fluctuations in the intensity of the filtered field *I*_Γ_(*ω*_*i*_) = 〈*I*_Γ_(*ω*_*i*_)〉 + *δI*_Γ_(*ω*_*i*_) for the two frequencies shown at the top panel and two values of *γ*, *γ* ≈ 8 × 10^−3^Γ (solid lines, middle panel), and *γ* ≈ 0.8Γ (dashed lines, bottom panel). The corresponding values of 
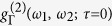
 are 0.97 and 0.65 resp. In the middle-panel case, where 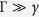, the anticorrelations in the noise become exact.

To the best of our knowledge, these features portray the first evidence of a HBT effect generalized to the full frequency-frequency domain. We now discuss these results in detail. Bunching in the diagonal line (corresponding to filters of equal frequency) is the well known feature of spectral filtering from a single peak^32^. From a classical point of view, this can be understood with the particular case of a quasi-monochromatic field *E*(*t*) that has a finite bandwith given by a phase diffusion process:


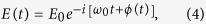


where *ϕ*(*t*) is a stochastic function that evolves, for instance, according to a random walk (see Fig. 3A). Such an errand phase allows for the line broadening. As is clear from Eq. (3), the frequency-filtered field is obtained by summing the field to itself at different times. If phase diffusion is present, this corresponds to the superposition of fields with random phases, which is analogous to the description of a thermal field. Consequently, such a superposition of fields of equal frequency but different phase produces interferences that wildly oscillate in a chaotic intensity profile, resulting in fluctuations in the intensity of the filtered field *I*_*ω*,Γ_ that satisfy:


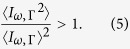


This is well known textbook material^33^. Interpreted in terms of photons, the underlying particles thus tend to “clump” together, and increase the spacing between their arrival time, which gives rise to the bunching effect.

We have just seen how phase noise is thus converted into intensity noise by frequency-filtering (see Fig. 3B,C). In a related but subtler way—which is the novel feature that we report—different frequencies open the possibility for anticorrelations. This remains true at the single particle level, as is demonstrated by our experiment, with anticorrelations between photons of different colors. Since the effect is linked to the aforementioned conversion of phase noise into amplitude noise by filtering, we can keep the paradigmatic case of a quasi-monochromatic field, that only has phase noise. On physical grounds, one expects that a field with a stabilized Poynting vector (in which the uncertainty in the number of photons detected in a certain time window is given by the shot noise) cannot yield in average more photon-counting events per unit time when spectrally resolved than it does without being frequency-filtered. Therefore, the detection of a clump of photons of some frequency in a small time window—in which photons are detected as random events prior filtering—must lower the probability of detecting photons at other, different frequencies, in order for the total rate of detected photons to be preserved. The anticorrelation we observe can therefore be interpreted as a consequence of energy conservation acting together with the HBT effect, that yields bunching of indistinguishable photons of equal frequencies. The photons on the detector, even if unrelated in the first place, cannot afford to remain so when frequency-filtered.

This argument is verified by explicit computation of Eq. (1) applied to the field of Eq. (4), assuming random walk dynamics for the phase such that 〈*e*^*i*[*ϕ*(*t*)−*ϕ*(*t*−*τ*)]^〉 = *e*^−γ|*τ*|^, 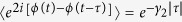. Here *γ* corresponds to the natural linewidth of the field due to phase fluctuation and *γ*_2_ is the fourth order correlation constant that, for a phase diffusing field, is given by *γ*_2_ = 4*γ*, as will be assumed from now on. The analytical expression for the frequency resolved correlation function at zero delay can be found exactly (the details of the calculation are given in Section I of the Supplemental Material):


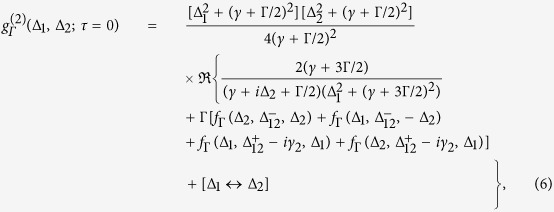


where Δ_*i*_ ≡ *ω*_0_ − *ω*_*i*_, 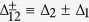 and *f*_Γ_(*ω*_1_, *ω*_2_, *ω*_3_) ≡ 1/[(*iω*_1_ + *γ* + Γ/2) (*iω*_2_ + Γ) (*iω*_3_ + *γ* + 3Γ/2)]. This expression reflects the same structure of correlations and anticorrelations observed in the experiment, as depicted in Fig. 3D,E, where it is shown to fit very well the experimental data. The main assumption behind this equation—that the unfiltered field has negligible amplitude fluctuations—is closely met in the experiment, in which the high coherence degree of the light emitted by the polaritons around the condensation threshold allows to unambiguously observe the anticorrelations. Just as the autocorrelations of Hanbury Brown for radio-waves of same frequencies (with no filtering), these anticorrelations of the filtered signal are obvious even to the naked eye, as can be seen in Fig. 3F, showing the intensity fluctuations of the simulated phase-diffusing field after frequency filtering. Surprisingly, such anticorrelations in the noise can even become exact, when the filter linewidth becomes much larger than the natural linewidth of the field (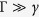). This is proved in Section I of the Supplemental Material. In this case, although 
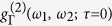
 gets closer to one (converging to the “unfiltered” result), the smaller fluctuations *δI*_Γ_(*ω*_*i*_) around the mean value *I*_Γ_(*ω*_*i*_) = 〈*I*_Γ_(*ω*_*i*_)〉 + *δI*_Γ_(*ω*_*i*_) tend to become perfectly anticorrelated for frequencies in opposite sides of the spectrum, *δI*_Γ_(*ω*_0_ − *ω*) ≈ − *δI*_Γ_(*ω*_0_ + *ω*), as can be observed in the middle panel of Fig. 3F.

Describing this effect from the quantum/particle point of view poses more difficulties. Since the 2PS is a dynamical observable, one cannot obtain the frequency-filtered photon correlations from a given quantum state without also including information about its dynamics, unlike the case without frequency-filtering where the knowledge of the diagonal elements of the density matrix is sufficient to compute 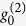. Such differences are discussed from a more technical point of view in Section II of the Supplemental Material. This makes the formulation of a general statement on the relation between a quantum state and the 2PS an ill-defined task. We consider for that purpose a simple situation in which an arbitrary quantum state given by the density matrix *ρ*(0) is left to decay from a source to a continuum of modes under spontaneous emission with a rate *γ*_*a*_ and also with a pure dephasing rate *γ*_*ϕ*_, thus eliminating every possible dynamics except the essential one that brings photons from the source to the detector, along with some dephasing mechanism. Therefore, the resulting master equation is given by 
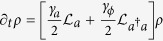
, where 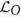 denotes the usual Lindblad term, 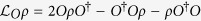. We have obtained the analytical expression of the normalized correlations at different frequencies integrated in time, which takes the form:


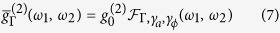


with 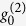 the zero delay second-order correlation function of the initial state and 
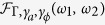
 a form factor (see Figs 1C and Fig. 3E and Section III of the Supplemental Material for the analytical expression), independent of this state, that reproduces exactly the features observed in the experiment and therefore captures the essence of this extension of the HBT effect. The wide range of frequencies used in Fig. 1C allows to show the nontrivial shape of the anticorrelations along the antidiagonal line (*ω*, − *ω*), featuring a minimum approximately at the point where the total filtered intensity is maximum without a considerable overlapping of the filters. The result alos shows that some dephasing mechanism is essential for the manifestation of the phenomenon (as it is for the standard HBT effect). This is directly implied in the classical picture and consistently confirmed in the quantum calculation, since when the dephasing rate *γ*_*ϕ*_ is equal to zero, 
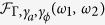
 is equal to one, and therefore featureless. This also explains why the coherent part of the resonance fluorescence spectrum, not subjected to dephasing, does not present this feature while the incoherent part does (see Fig. 7 of ref. 23). Consistently with the classical analysis based on a stochastic field of Eq. (4), the quantum calculation shows that in the particular case when the unfiltered field has no intensity fluctuations, i.e., 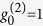, the filtered field displays anticorrelations. Both general analyses (classical and quantum), together with the experiment and the theoretical characterization of the steady state emission of our specific system, complete our description of the effect.

**Figure 4:**
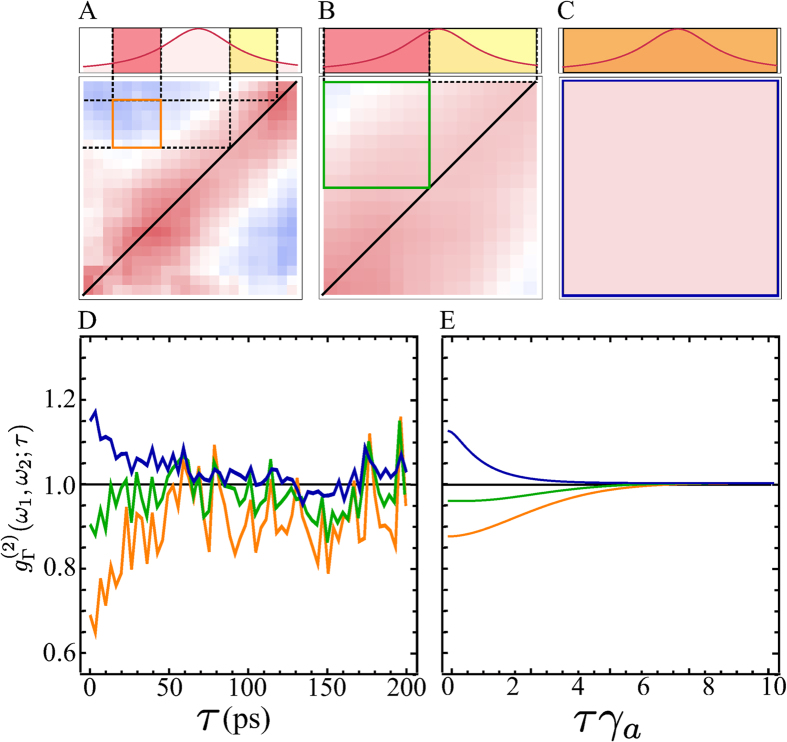
Two-photon correlation landscapes 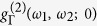 as a function of the filter width Γ, from a fraction of the peak, Γ = 74.1 µeV (**A**), roughly half-peak width, Γ = 158.8 µeV (**B**), to full-peak filtering (**C**), corresponding to standard auto-correlations. The position of the two filters is shown explicitly on the spectral line as the red and yellow windows (orange when overlapping). Panels D,E describe the experiment and the theory from the condensation model, respectively. The values employed for the theoretical simulation are Γ = {0.5, 0.75, 1.5}*γ*_*a*_, giving a good description of the experiment for *γ*_*a*_ ≈ 200 µeV. This value is also consistent with the one extracted from the fitting of Eq. (6), see caption of Fig. 3.

Another fundamental feature of the theory^31^ is that correlations depend on the frequency windows that select which photons are correlated. Smaller windows lead to stronger correlations but, again, at the price of a smaller signal. While it does not correspond exactly to a change in the width of the filter, the effect is neatly illustrated by changing the number of pixels of the streak camera that we associate to a given frequency. In Fig. 4, we show the dependence of the 2PS on the size of the frequency windows for a point that features antibunching. When the frequency window is very large (c), Γ ≫ *γ*_*a*_, both the experimental and theoretical 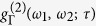 recover as expected the results of standard photon correlations which has always been reported to be larger than 1 for this kind of systems^34^,^35^,^36^. As the size of the frequency window decreases, the system shows a transition from bunching to antibunching (a–c), demonstrating how the statistics of coloured photons can be easily tuned externally. Such results, that generalize the Hanbury Brown–Twiss effect to exhibit correlations of different types depending on the energies—are of fundamental interest, but are also of technological importance. Indeed, the main observable—the frequency-resolved correlation function—is of increasing importance in quantum-optical technologies. Spectrally-resolved photon counting measurements can be a useful tool in non-linear spectroscopy, able to measure ultrafast dynamics^37^. Applications for measuring fluctuations through detection of single photons in time and frequency with picosecond resolution in spectral diffusion problems^20^ should also benefit from both our findings and experimental setup. Our results and methods are also of importance for the study of quantum systems with more complex dynamics beyond merely spontaneous emission and dephasing^23^,^31^. In cases of strongly correlated emission, virtual processes^38^,^39^ result in 2PS with strong geometric features, such as antidiagonals or circles of correlations^23^,^24^. Such rich landscapes of photon correlations, inherited from the system’s quantum dynamics, are otherwise lost by disregarding the frequencies. The results presented here provide the backbone for more general schemes that, for practical purposes such as distillation^40^ or Purcell enhancement^38^,^41^, can be used to power quantum technology. Like Purcell did in his pioneering interpretation of the HBT effect^7^, we conjecture a counterpart for fermion correlations^42^, namely, with an orthogonal profile: antibunching on the diagonal and bunching on the antidiagonal; this question, that could be investigated for instance in transport experiments with electrons^43^, is however outside the scope of this text and its field of research, and is left to experimental and theoretical colleagues in other disciplines.

## Conclusion

We report the measurement of anticorrelations between individual photons emitted from a ensemble of polaritons under continuous pumping. We have demonstrated that this phenomenon is a fundamental result that generalizes the Hanbury Brown–Twiss effect for color correlations, and is therefore linked to the bosonic nature of photons. We have introduced a novel experimental technique that allows to measure correlations in time and energy between individual photons, demonstrating that both the concept and technique of color correlations are sound and ripe to be deployed in a large range of quantum optical systems, with prospects of accessing further classes of quantum correlations^44^,^45^, optimising those already known^39^,^40^, or analysing problems such as spectral diffusion at new levels of precision^20^.

## Methods

### Materials.

The sample used for this measurement was a high-Q planar microcavity (Q = 100 000) containing 12 GaAs quantum wells placed at three antinode positions of the electrical field. The front (back) mirror consists of 34 (40) pairs of AlAs/Al_0.2_Ga_0.8_As layers. The detuning was slightly negative, with the cavity component at 1610 meV, the excitonic at 1611 meV and a Rabi splitting of 16 meV. The single-mode Ti:sapphire ring laser was exciting offresonantly at 1710 meV and at a power of 40 mW set to the threshold of the condensate. The pumping was at normal incidence with a spot size of 20 *µ*m of diameter. Thanks to a double electronic synchronization, an additional “slow” sweeping in time is performed also in the horizontal direction, thus recording multiple sweeps per frame: in this way the number of events detected in each frame is increased of about one order of magnitude, allowing the detection of a large statistics of events in a relatively short measurement time, going beyond the limits imposed by the electronics speed of CCD devices.

## Additional Information

**How to cite this article**: Silva, B. *et al*. The colored Hanbury Brown–Twiss effect. *Sci. Rep.* **6**, 37980; doi: 10.1038/srep37980 (2016).

**Publisher's note:** Springer Nature remains neutral with regard to jurisdictional claims in published maps and institutional affiliations.

## Supplementary Material

Supplementary Material

## References

[b1] Hanbury Brown R. & Twiss R. Q. A test of a new type of stellar interferometer on Sirius. Nature 178, 1046 (1956).

[b2] Hanbury Brown R., Jennison R. C. & Gupta M. K. D. Apparent angular sizes of discrete radio sources: Observations at jodrell bank, manchester. Nature 170, 1061 (1952).

[b3] Hanbury Brown R. & Twiss R. Q. Correlation between photons in two coherent beams of light. Nature 177, 27 (1956).

[b4] Glauber R. J. Coherent and incoherent states of the radiation field. Phys. Rev. 131, 2766 (1963).

[b5] Brown R. H. Boffin: A Personal Story of the Early Days of Radar, Radio Astronomy and Quantum Optics (CRC Press, 1991).

[b6] Baym G. The physics of Hanbury Brown–Twiss intensity interferometry: from stars to nuclear collisions. Acta Physica Polonica B 29, 1839 (1998).

[b7] Purcell E. M. The question of correlation between photons in coherent light rays. Nature 178, 1449 (1956).

[b8] Glauber R. J. Nobel lecture: One hundred years of light quanta. Rev. Mod. Phys. 78, 1267 (2006). 10.1002/cphc.200600329 16888746

[b9] Stevenson R. M. . A semiconductor source of triggered entangled photon pairs. Nature 439, 179 (2006). 16407947 10.1038/nature04446

[b10] Jeltes T. . Comparison of the Hanbury Brown–Twiss effect for bosons and fermions. Nature 445, 402 (2007). 17251973 10.1038/nature05513

[b11] Ekert A. K. Quantum cryptography based on Bell’s theorem. Phys. Rev. Lett. 67, 661 (1991). 10044956 10.1103/PhysRevLett.67.661

[b12] Cohen-Tannoudji C. & Reynaud S. Atoms in strong light-fields: Photon antibunching in single atom fluorescence. Phil. Trans. R. Soc. Lond. A 293, 223 (1979).

[b13] Dalibard J. & Reynaud S. Correlation signals in resonance fluorescence: interpretation via photon scattering amplitudes. J. Phys. France 44, 1337 (1983).

[b14] Knöll L. & Weber G. Theory of *n*-fold time-resolved correlation spectroscopy and its application to resonance fluorescence radiation. J. Phys. B.: At. Mol. Phys. 19, 2817 (1986).

[b15] Nienhuis G. Spectral correlations in resonance fluorescence. Phys. Rev. A 47, 510 (1993). 9908943 10.1103/physreva.47.510

[b16] Kamide K., Iwamoto S. & Arakawa Y. Eigenvalue decomposition method for photon statistics of frequency-filtered fields and its application to quantum dot emitters. Phys. Rev. A 92, 033833 (2015).

[b17] Akopian N. . Entangled photon pairs from semiconductor quantum dots. Phys. Rev. Lett. 96, 130501 (2006). 16711973 10.1103/PhysRevLett.96.130501

[b18] Hennessy K. . Quantum nature of a strongly coupled single quantum dot–cavity system. Nature 445, 896 (2007). 17259971 10.1038/nature05586

[b19] Kaniber M. . Investigation of the nonresonant dot-cavity coupling in two-dimensional photonic crystal nanocavities. Phys. Rev. B 77, 161303(R) (2008).

[b20] Sallen G. . Subnanosecond spectral diffusion measurement using photon correlation. Nat. Photon. 4, 696 (2010).

[b21] Ulhaq A. . Cascaded single-photon emission from the Mollow triplet sidebands of a quantum dot. Nat. Photon. 6, 238 (2012).

[b22] Deutsch Z., Schwartz O., Tenne R., Popovitz-Biro R. & Oron D. Two-color antibunching from band-gap engineered colloidal semiconductor nanocrystals. Nano Lett. 12, 2948 (2012). 22533783 10.1021/nl300638t

[b23] González-Tudela A., Laussy F. P., Tejedor C., Hartmann M. J. & del Valle E. Two-photon spectra of quantum emitters. New J. Phys. 15, 033036 (2013).

[b24] Peiris M. . Two-color photon correlations of the light scattered by a quantum dot. Phys. Rev. B 91, 195125 (2015).

[b25] González-Tudela A., del Valle E. & Laussy F. P. Optimization of photon correlations by frequency filtering. Phys. Rev. A 91, 043807 (2015).

[b26] Kavokin A., Baumberg J. J., Malpuech G. & Laussy F. P. Microcavities 2 edn (Oxford University Press, 2011).

[b27] Deng H., Weihs G., Santori C., Bloch J. & Yamamoto Y. Condensation of semiconductor microcavity exciton polaritons. Science 298, 199 (2002). 12364801 10.1126/science.1074464

[b28] Aßmann M., Veit F., Bayer M., van der Poel M. & Hvam J. M. Higher-order photon bunching in a semiconductor microcavity. Science 325, 297 (2009). 19608912 10.1126/science.1174488

[b29] Adiyatullin A. F. . Temporally resolved second-order photon correlations of exciton-polariton bose-einstein condensate formation. Appl. Phys. Lett. 107, 221107 (2015).

[b30] Wiersig J. . Direct observation of correlations between individual photon emission events of a microcavity laser. Nature 460, 245 (2009). 19587766 10.1038/nature08126

[b31] del Valle E., González-Tudela A., Laussy F. P., Tejedor C. & Hartmann M. J. Theory of frequency-filtered and time-resolved *n*-photon correlations. Phys. Rev. Lett. 109, 183601 (2012). 23215277 10.1103/PhysRevLett.109.183601

[b32] Centeno Neelen R., Boersma D. M., van Exter M. P., Nienhuis G. & Woerdman J. P. Spectral filtering within the Schawlow-Townes linewidth of a semiconductor laser. Phys. Rev. Lett. 69, 593 (1992). 10046981 10.1103/PhysRevLett.69.593

[b33] Loudon R. The quantum theory of light, 3 edn (Oxford Science Publications, 2000).

[b34] Love A. P. D. . Intrinsic decoherence mechanisms in the microcavity polariton condensate. Phys. Rev. Lett. 101, 067404 (2008). 18764503 10.1103/PhysRevLett.101.067404

[b35] Kasprzak J. . Second-order time correlations within a polariton Bose–Einstein condensate in a CdTe microcavity. Phys. Rev. Lett. 100, 067402 (2008). 18352514 10.1103/PhysRevLett.100.067402

[b36] Aßmann M. . From polariton condensates to highly photonic quantum degenerate states of bosonic matter. Proc. Natl. Acad. Sci. 108, 1804 (2011). 21245353 10.1073/pnas.1009847108 PMC3033249

[b37] Dorfman K. E., Schlawin F. & Mukamel S. Nonlinear optical signals and spectroscopy with quantum light. arXiv:1605.06746 (2016).

[b38] Sánchez Muñoz C. . Emitters of *N*-photon bundles. Nat. Photon. 8, 550 (2014). 10.1038/nphoton.2014.114 PMC4083484 25013456

[b39] Sánchez Muñoz C., del Valle E., Tejedor C. & Laussy F. Violation of classical inequalities by photon frequency filtering. Phys. Rev. A 90, 052111 (2014).

[b40] del Valle E. Distilling one, two and entangled pairs of photons from a quantum dot with cavity QED effects and spectral filtering. New J. Phys. 15, 025019 (2013).

[b41] Sánchez Muñoz C., Laussy F. P., Tejedor C. & del Valle E. Enhanced two-photon emission from a dressed biexciton. New J. Phys. 17, 123021 (2015).

[b42] Henny M. . The fermionic Hanbury Brown and Twiss experiment. Science 284, 296 (1999). 10195890 10.1126/science.284.5412.296

[b43] Bocquillon E. . Electron quantum optics in ballistic chiral conductors. Annalen der Physik 526, 1 (2014).

[b44] Koch M. . Three-photon correlations in a strongly driven atom-cavity system. Phys. Rev. Lett. 107, 023601 (2011). 21797605 10.1103/PhysRevLett.107.023601

[b45] Rundquist A. . Nonclassical higher-order photon correlations with a quantum dot strongly coupled to a photonic-crystal nanocavity. Phys. Rev. A 90, 023846 (2014).

