# Supplementary material for: The colored Hanbury Brown–Twiss effect
Source: Sci Rep. 2016 Dec 6;6:37980. doi: 10.1038/srep37980 (PMC5138626; doi:10.1038/srep37980)
Supplement: Supplementary Material [file srep37980-s1.pdf]

The colored Hanbury Brown–Twiss effect

Blanca Silva[1,2], C. Sánchez Muñoz[2], D. Ballarini[1], A. González Tudela[3], M. de Giorgi[1], G. Gigli[1], K. W. West[4], L. Pfeiffer[4], E. del Valle[2], D. Sanvitto[1], and F. P. Laussy[2,5,*]

[1]CNR NANOTEC–Institute of Nanotechnology, Via Monteroni, 73100 Lecce, Italy
[2]Departamento de Física Teórica de la Materia Condensada and Condensed Matter Physics Center (IFIMAC)
[3]Max–Planck Institut für Quantenoptik, 85748 Garching, Germany
[4]Department of Electrical Engineering, Princeton University, Princeton, New Jersey 08544, USA
[5]Russian Quantum Center, Novaya 100, 143025 Skolkovo, Moscow Region, Russia
[*]fabrice.laussy@gmail.com

ABSTRACT

In this theoretical Supplementary Material, we provide the details of the derivations for the results given in the main text.

I. Calculation of $g_\Gamma^{(2)}(\omega_1,\omega_2,\tau)$ for a phase diffusing field

In this section, we give the details of the calculation of the frequency-resolved correlation function for a classical stochastic field. Such a phase-diffusing field, but with otherwise stabilized intensity (describing a coherent field), reads:

$$E(t) = E_0 e^{-i[\omega_0 t + \phi(t)]}\,, \tag{1}$$

where the phase is a random variable that is considered to undergo a random walk evolution. The phase difference $\Delta\phi(\tau) = \phi(t+\tau) - \phi(t)$ has the following properties:

$$\begin{aligned}
\langle \Delta\phi(\tau)\rangle &= 0\,,\\
\langle \Delta\phi(\tau)^2\rangle &= 2\gamma|\tau|\,,\\
\langle e^{i[\phi(t)-\phi(t-\tau)]}\rangle &= e^{-\gamma|\tau|}\,,\\
\langle e^{2i[\phi(t)-\phi(t-\tau)]}\rangle &= e^{-\gamma_2|\tau|}\,.
\end{aligned} \tag{2}$$

In the case of a phase diffusing field, the fourth order correlation constant γ_2 is given by $\gamma_2 = 4\gamma$. However, we keep γ_2 in the expressions to account for other possible models of phase noise, like the phase-jump model[1], in which case $\gamma_2 = \gamma$. These properties allow us to compute the frequency resolved second-order correlation function:

$$g_\Gamma^{(2)}(\omega_1,\omega_2,\tau) = \frac{\langle E^+_{\omega_2,\Gamma}(t+\tau)E_{\omega_2,\Gamma}(t+\tau)E^+_{\omega_1,\Gamma}(t)E_{\omega_1,\Gamma}(t)\rangle}{\langle E^+_{\omega_2,\Gamma}(t+\tau)E_{\omega_2,\Gamma}(t+\tau)\rangle\langle E^+_{\omega_1,\Gamma}(t)E_{\omega_1,\Gamma}(t)\rangle}\,, \tag{3}$$

where $E_{\omega,\Gamma}(t)$ describes the field filtered at frequency ω with a filter linewidth Γ:

$$E_{\omega,\Gamma}(t) = \frac{\Gamma}{2}\int_0^\infty e^{-i\omega t'}e^{-\Gamma t'/2}E(t-t')\,dt'. \tag{4}$$

This calculation is done with the properties listed in Eq. (2). The numerator of Eq. (3), that we denote $G_\Gamma^{(2)}(\omega_1,\omega_2,\tau)$, is given by the following quadruple integral:

$$\begin{aligned}
G_\Gamma^{(2)}(\omega_1,\omega_2,\tau) &= \left(\frac{\Gamma}{2}\right)^4 \iiiint_0^\infty \prod_{i=1}^4 dt_i\, e^{i\omega_2(t_4-t_3)}e^{i\omega_1(t_2-t_1)}e^{-\Gamma(t_1+t_2+t_3+t_4)/2}\langle E^*(t-t_4+\tau)E(t-t_3+\tau)E^*(t-t_2)E(t-t_1)\rangle\\
&= E_0^4\left(\frac{\Gamma}{2}\right)^4 \iiiint_0^\infty \prod_{i=1}^4 dt_i\, e^{-i\Delta_1(t_2-t_1)-i\Delta_2(t_4-t_3)}e^{-\Gamma(t_1+t_2+t_3+t_4)/2}\langle e^{-i[\phi(t-t_1)-\phi(t-t_2)+\phi(t-t_3+\tau)-\phi(t-t_4+\tau)]}\rangle\,,
\end{aligned} \tag{5}$$

where $\Delta_i \equiv \omega_0 - \omega_i$. Defining $t_1' \equiv t - t_1$, $t_2' \equiv t - t_2$, $t_3' \equiv t - t_3 + \tau$ and $t_4' \equiv t - t_4 + \tau$, the statistical average in the last line of (5) takes the form $\langle e^{i[\phi(t_1')-\phi(t_2')+\phi(t_3')-\phi(t_4')]}\rangle$. The exponent can be written in term of phase differences $\Delta\phi(\tau)$ in two

possible ways, $\langle e^{i[\Delta\phi(t_1'-t_2')+\Delta\phi(t_3'-t_4')]}\rangle$ or $\langle e^{i[\Delta\phi(t_1'-t_4')+\Delta\phi(t_3'-t_2')]}\rangle$. For a given set of values for t_1', t_2', t_3' and t_4', the choice between both options must be made such that the two $\Delta\phi$ are defined in non-overlapping time intervals, making them statistically independent. This allows to factorize the exponential and use Eq. (2) to evaluate the statistical averages. Figure 1 depicts the three possible configurations that exist depending on the values of t_i'. Panel c shows the particular case $t_4', t_2' < t_1', t_3'$ that requires the introduction of a third time interval to avoid overlapping; this is the case that will invoke the last equation in Eq. (2), involving the fourth order correlation constant γ_2. Since the integrand has to be written differently depending on the values of the t_i', one needs to split the integral in the 24 possible domains. Half of these integrals are the complex conjugate of the other half, yielding 12 independent terms, defined in the domains:

$$
\begin{aligned}
\mathrm{I}: &\quad t_2' < t_1' < t_3' < t_4' &\overset{*}{\leftrightarrow}&\quad t_1' < t_2' < t_4' < t_3'\,,\\
\mathrm{II}: &\quad t_2' < t_3' < t_1' < t_4' &\overset{*}{\leftrightarrow}&\quad t_1' < t_4' < t_2' < t_3'\,,\\
\mathrm{III}: &\quad t_4' < t_3' < t_1' < t_2' &\overset{*}{\leftrightarrow}&\quad t_3' < t_4' < t_2' < t_1'\,,\\
\mathrm{IV}: &\quad t_4' < t_1' < t_3' < t_2' &\overset{*}{\leftrightarrow}&\quad t_3' < t_2' < t_4' < t_1'\,,\\
\mathrm{V}: &\quad t_2' < t_1' < t_4' < t_3' &\overset{*}{\leftrightarrow}&\quad t_1' < t_2' < t_3' < t_4'\,,\\
\mathrm{VI}: &\quad t_2' < t_3' < t_4' < t_1' &\overset{*}{\leftrightarrow}&\quad t_1' < t_4' < t_3' < t_2'\,,\\
\mathrm{VII}: &\quad t_4' < t_3' < t_2' < t_1' &\overset{*}{\leftrightarrow}&\quad t_3' < t_4' < t_1' < t_2'\,,\\
\mathrm{VIII}: &\quad t_4' < t_1' < t_2' < t_3' &\overset{*}{\leftrightarrow}&\quad t_3' < t_2' < t_1' < t_4'\,,\\
\mathrm{IX}: &\quad t_2' < t_4' < t_1' < t_3' &\overset{*}{\leftrightarrow}&\quad t_1' < t_3' < t_2' < t_4'\,,\\
\mathrm{X}: &\quad t_2' < t_4' < t_3' < t_1' &\overset{*}{\leftrightarrow}&\quad t_1' < t_3' < t_2' < t_4'\,,\\
\mathrm{XI}: &\quad t_4' < t_2' < t_1' < t_3' &\overset{*}{\leftrightarrow}&\quad t_3' < t_1' < t_2' < t_4'\,,\\
\mathrm{XII}: &\quad t_4' < t_2' < t_3' < t_1' &\overset{*}{\leftrightarrow}&\quad t_3' < t_1' < t_4' < t_2'\,.
\end{aligned}
\tag{6}
$$

By denoting the non-overlapping time differences τ_i and making a change of variables, the corresponding integrals read:

$$
\begin{aligned}
I_{\mathrm{I}} &= C\int_0^\infty dt_2 \int_{t_2}^0 d\tau_1 \int_0^{t_2-\tau_1+\tau} dt_3 \int_{t_3}^0 d\tau_2\, e^{i(\Delta_2\tau_2-\Delta_1\tau_1)} e^{-\Gamma(2t_2+2t_3-\tau_1-\tau_2)/2-\gamma(\tau_1+\tau_2)},\\
I_{\mathrm{II}} &= C\int_0^\infty dt_2 \int_{t_2}^0 d\tau_1 \int_0^{t_2-\tau_1} dt_1 \int_{t_1+\tau}^0 d\tau_2\, e^{i\Delta_1(t_1-t_2)+i\Delta_2(\tau_2-\tau_1+t_2-t_1)} e^{-\Gamma(2t_2-\tau_1+2t_1-\tau_2+2\tau)/2-\gamma(\tau_1+\tau_2)},\\
I_{\mathrm{III}} &= C\int_\tau^\infty dt_4 \int_{t_4-\tau}^0 d\tau_1 \int_0^{t_4-\tau_1-\tau} dt_1 \int_{t_1}^0 d\tau_2\, e^{i(\Delta_1\tau_2-\Delta_2\tau_1} e^{-\Gamma(2t_4+2t_1-\tau_1-\tau_2)/2-\gamma(\tau_1+\tau_2)},\\
I_{\mathrm{IV}} &= C\int_\tau^\infty dt_4 \int_{t_4-\tau}^0 d\tau_1 \int_\tau^{t_4-\tau_1} dt_3 \int_{t_3-\tau}^0 d\tau_2\, e^{i\Delta_2(t_3-t_4)+i\Delta_1(\tau_2-\tau_1+t_4-t_3)} e^{-\Gamma(2t_4+2t_3-\tau_1-\tau_2-2\tau)/2-\gamma(\tau_1+\tau_2)},\\
I_{\mathrm{V}} &= C\int_0^\infty dt_2 \int_{t_2}^0 d\tau_1 \int_0^{t_2-\tau_1+\tau} dt_4 \int_{t_4}^0 d\tau_2\, e^{-i(\Delta_1\tau_1+\Delta_2\tau_2)} e^{-\Gamma(2t_2+2t_4-\tau_1-\tau_2)/2-\gamma(\tau_1+\tau_2)},\\
I_{\mathrm{VI}} &= C\int_0^\infty dt_2 \int_{t_2}^0 d\tau_1 \int_\tau^{t_2-\tau_1+\tau} dt_4 \int_{t_4-\tau}^0 d\tau_2\, e^{-i\Delta_1(t_2-t_4+\tau+\tau_2)-i\Delta_2(t_4-t_2-\tau+\tau_1)} e^{-\Gamma(2t_2+2t_4-\tau_1-\tau_2)/2-\gamma(\tau_1+\tau_2)},\\
I_{\mathrm{VII}} &= C\int_\tau^\infty dt_4 \int_{t_4-\tau}^0 d\tau_1 \int_0^{t_4-\tau_1-\tau} dt_2 \int_{t_2}^0 d\tau_2\, e^{-i\Delta_1\tau_2-i\Delta_2\tau_1} e^{-\Gamma(2t_4+2t_2-\tau_1-\tau_2)/2-\gamma(\tau_1+\tau_2)},\\
I_{\mathrm{VIII}} &= C\int_\tau^\infty dt_4 \int_{t_4-\tau}^0 d\tau_1 \int_0^{t_4-\tau_1-\tau} dt_2 \int_{t_2+\tau}^0 d\tau_2\, e^{-i\Delta_1(t_2-t_4+\tau+\tau_1)-i\Delta_2(t_4-t_2-\tau+\tau_2)} e^{-\Gamma(2t_4+2t_2-\tau_1-\tau_2)/2-\gamma(\tau_1+\tau_2)},\\
I_{\mathrm{IX}} &= -C\int_0^\infty dt_2 \int_{t_2}^0 d\tau_1 \int_{t_2-\tau_1}^0 d\tau_2 \int_{t_2-\tau_1-\tau_2+\tau}^0 d\tau_3\, e^{-i\Delta_1(\tau_1+\tau_2)-i\Delta_2(\tau_2+\tau_3)} e^{-\Gamma(4t_2-3\tau_1-2\tau_2-\tau_3+2\tau)/2-\gamma(\tau_1+\tau_3)-\gamma_2\tau_2},\\
I_{\mathrm{X}} &= -C\int_0^\infty dt_2 \int_{t_2}^0 d\tau_1 \int_{t_2-\tau_1}^0 d\tau_2 \int_{t_2-\tau_1-\tau_2}^0 d\tau_3\, e^{-i\Delta_1(\tau_1+\tau_2+\tau_3)-i\Delta_2\tau_2} e^{-\Gamma(4t_2+2\tau-3\tau_1-2\tau_2-\tau_3)/2-\gamma(\tau_1+\tau_3)-\gamma_2\tau_2},\\
I_{\mathrm{XI}} &= -C\int_\tau^\infty dt_4 \int_{t_4-\tau}^0 d\tau_1 \int_{t_4-\tau-\tau_1}^0 d\tau_2 \int_{t_4-\tau_1-\tau_2}^0 d\tau_3\, e^{-i\Delta_1\tau_2-i\Delta_2(\tau_1+\tau_2+\tau_3)} e^{-\Gamma(4t_4-2\tau-3\tau_1-2\tau_2-\tau_3)/2-\gamma(\tau_1+\tau_3)-\gamma_2\tau_2},\\
I_{\mathrm{XII}} &= -C\int_\tau^\infty dt_4 \int_{t_4-\tau}^0 d\tau_1 \int_{t_4-\tau-\tau_1}^0 d\tau_2 \int_{t_4-\tau_1-\tau_2-\tau}^0 d\tau_3\, e^{-i\Delta_1(\tau_2+\tau_3)-i\Delta_2(\tau_1+\tau_2)} e^{-\Gamma(4t_4-2\tau-3\tau_1-2\tau_2-\tau_3)/2-\gamma(\tau_1+\tau_3)-\gamma_2\tau_2},
\end{aligned}
\tag{7}
$$

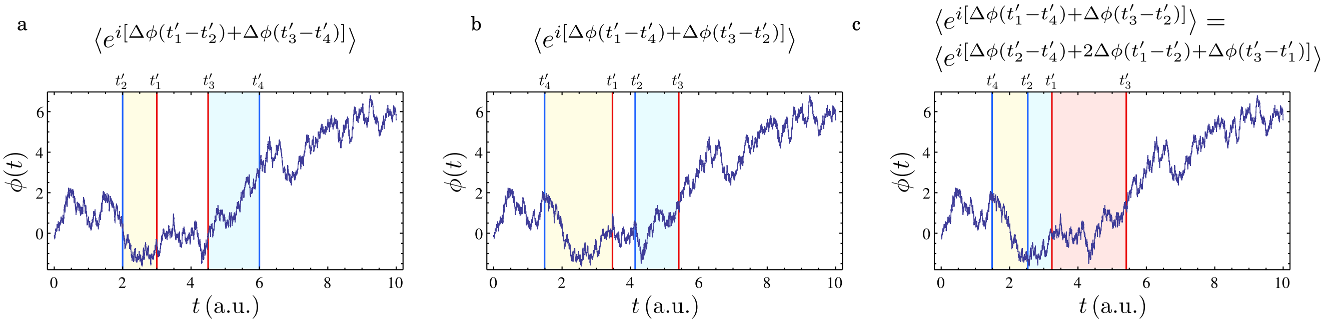

Figure 1. Examples of the possible integration domains for the fluctuating phase corresponding to three possible exponents that appear in the integrals: (a) Domain I, (b) Domain VIII and (c) Domain XI. In domains IX–XII, the exponent must be written as three phase differences to ensure they are statistically uncorrelated.

with $C = E_0^4\left(\frac{\Gamma}{2}\right)^4$. Finally, $G_\Gamma^{(2)}(\omega_1,\omega_2,\tau)$ is given by:

$$
\begin{aligned}
&G_\Gamma^{(2)}(\omega_1,\omega_2,\tau) = 2\mathrm{Re}\sum_{i=\mathrm{I}}^{\mathrm{XII}} I_i = \\
&\frac{E_0^4}{16}\Gamma^2\mathrm{Re}\left\{\frac{2ie^{\tau\left(-\gamma-\frac{\Gamma}{2}-i\Delta_2\right)}\left(\gamma_2\left(\frac{i\Gamma}{2}+2\Delta_1-\Delta_2\right)+\left(\frac{\Gamma}{2}+i\Delta_1\right)(-i\Gamma+\Delta_1+\Delta_2)+\gamma(-3i\Gamma-i\gamma_2-\Delta_1+3\Delta_2)\right)\Gamma^2}{\left(\gamma+\frac{\Gamma}{2}+i\Delta_1\right)\left(\gamma-\frac{\Gamma}{2}+i\Delta_2\right)\left(\gamma+\frac{\Gamma}{2}+i\Delta_2\right)\left(\gamma+\frac{3\Gamma}{2}+i\Delta_2\right)(-i\Gamma-\Delta_1+\Delta_2)(-i\Gamma-i\gamma_2+\Delta_1+\Delta_2)}\right. \\
&+\frac{2e^{\tau\left(-\gamma-\frac{\Gamma}{2}+i\Delta_2\right)}\left(\gamma-\frac{\Gamma}{2}-i\Delta_1\right)\Gamma^2}{\left(\gamma+\frac{\Gamma}{2}+i\Delta_1\right)\left(\gamma+\frac{\Gamma}{2}-i\Delta_2\right)\left(\gamma+\frac{3\Gamma}{2}-i\Delta_2\right)\left(i\gamma-\frac{i\Gamma}{2}+\Delta_2\right)(i\Gamma-\Delta_1+\Delta_2)} \\
&+\frac{e^{-\Gamma\tau}\left(-\Gamma^3-\frac{1}{2}(4\gamma+i(3\Delta_1+\Delta_2))\Gamma^2+\frac{1}{2}(\Delta_1-\Delta_2)(2i\gamma-3i\gamma_2+\Delta_1+3\Delta_2)\Gamma+(\Delta_1-\Delta_2)(\gamma+i\Delta_2)(-i\gamma_2+\Delta_1+\Delta_2)\right)}{\left(\gamma+\frac{\Gamma}{2}+i\Delta_1\right)\left(\gamma-\frac{\Gamma}{2}+i\Delta_2\right)\left(\gamma+\frac{\Gamma}{2}+i\Delta_2\right)(-i\Gamma-\Delta_1+\Delta_2)(-i\Gamma-i\gamma_2+\Delta_1+\Delta_2)} \\
&+e^{-\Gamma\tau}\left[\frac{2\left(\gamma+\frac{3\Gamma}{2}\right)}{\left(\left(\gamma+\frac{3\Gamma}{2}\right)^2+\Delta_1^2\right)\left(\gamma+\frac{\Gamma}{2}+i\Delta_2\right)}+\frac{i\Gamma}{\left(\gamma+\frac{3\Gamma}{2}-i\Delta_1\right)(i\Gamma+\Delta_1-\Delta_2)\left(\gamma+\frac{\Gamma}{2}+i\Delta_2\right)}\right. \\
&+\frac{\Gamma}{\left(\gamma+\frac{\Gamma}{2}+i\Delta_1\right)\left(\gamma+\frac{3\Gamma}{2}+i\Delta_1\right)(\Gamma+\gamma_2+i(\Delta_1+\Delta_2))}+\frac{\Gamma}{\left(\gamma+\frac{3\Gamma}{2}+i\Delta_1\right)\left(\gamma+\frac{\Gamma}{2}+i\Delta_2\right)(\Gamma+\gamma_2+i(\Delta_1+\Delta_2))} \\
&+\left.\frac{\Gamma}{\left(\gamma+\frac{\Gamma}{2}+i\Delta_1\right)\left(\gamma+\frac{3\Gamma}{2}+i\Delta_1\right)(\Gamma+i(\Delta_1-\Delta_2))}\right]+\frac{2}{\left(\gamma+\frac{\Gamma}{2}+i\Delta_1\right)\left(\gamma+\frac{\Gamma}{2}-i\Delta_2\right)}+\frac{2}{\left(\gamma+\frac{\Gamma}{2}+i\Delta_1\right)\left(\gamma+\frac{\Gamma}{2}+i\Delta_2\right)} \\
&+\left.\frac{e^{-\Gamma\tau}(\Delta_2-\Delta_1)}{\left(\gamma+\frac{\Gamma}{2}+i\Delta_1\right)\left(-\gamma+\frac{\Gamma}{2}+i\Delta_2\right)(i\Gamma-\Delta_1+\Delta_2)}\right\}. \qquad (8)
\end{aligned}
$$

On the other hand, the denominator in Eq. (3) consists of the product of the mean intensities of the two filtered fields. This mean intensity is readily given by:

$$
\begin{aligned}
\langle E^+_{\omega_i,\Gamma}(t)E_{\omega_i,\Gamma}(t)\rangle = \langle I_{\omega_i,\Gamma}(t)\rangle &= E_0^2\frac{\Gamma^2}{4}\left(\int_0^\infty dt_1\int_0^{t_1} dt_2\, e^{i\Delta_i(t_1-t_2)-\Gamma(t_1+t_2)/2-\gamma(t_1-t_2)}+\right. \\
&\qquad\left.\int_0^\infty dt_2\int_0^{t_2} dt_1\, e^{i\Delta_i(t_1-t_2)-\Gamma(t_1+t_2)/2-\gamma(t_2-t_1)}\right) \\
&= E_0^2\frac{\Gamma^2}{2}\mathrm{Re}\int_0^\infty dt_1\int_0^{t_1} dt_2\, e^{(i\Delta_i-\gamma)(t_1-t_2)-\Gamma(t_1+t_2)/2} = E_0^2\frac{\Gamma}{2}\frac{\gamma+\Gamma/2}{\Delta_i^2+(\gamma+\Gamma/2)^2}. \qquad (9)
\end{aligned}
$$

Normalizing the expression of $G_\Gamma^{(2)}(\omega_1,\omega_2,\tau)$ given in Eq. (8) by the intensities in Eq. (9), we obtain the final expression for $g_\Gamma^{(2)}(\omega_1,\omega_2,\tau)$. At $\tau = 0$, this expression takes the more compact form presented in the main text.

An interesting limit, reported in Fig. 3f of the main text, occurs when the filter linewidth is much larger than the natural

linewidth of the field, $\Gamma \gg \gamma$. The intensity of the filtered field is then given by:

$$\begin{aligned} I_{\omega_i,\Gamma}(t) &= E_0^2 \frac{\Gamma^2}{2} \mathrm{Re} \int_0^t dt_1 \int_0^{t_1} dt_2\, e^{-\Gamma(t_1+t_2)/2} e^{i[\Delta_i(t_1-t_2)+\phi(t-t_1)-\phi(t-t_2)]} \\ &= E_0^2 \frac{\Gamma^2}{2} \int_0^t dt_1 \int_0^{t_1} dt_2\, e^{-\Gamma(t_1+t_2)/2} \cos\left[\Delta_i(t_1-t_2)+\Delta\phi(t_1,t_2,t)\right] \\ &= E_0^2 \frac{\Gamma^2}{2} \int_0^t dt_1 \int_0^{t_1} dt_2\, e^{-\Gamma(t_1+t_2)/2} \{\cos\left[\Delta_i(t_1-t_2)\right] \cos[\Delta\phi(t_1,t_2,t)] \\ &\qquad - \sin[\Delta_i(t_1-t_2)] \sin[\Delta\phi(t_1,t_2,t)]\}\,, \end{aligned} \tag{10}$$

where $\Delta\phi(t_1,t_2,t) = \phi(t-t_1) - \phi(t-t_2)$. If $\Gamma \gg \gamma$, the timescale given by the filter linewidth is much shorter than the natural timescale of the filtered field, and we can assume $\Delta\phi(t_1,t_2,t) \ll 1$ for those values of t_1 and t_2 where the integrand is non-negligible. By expanding to first order in $\Delta\phi(t_1,t_2,t)$, we obtain:

$$I_{\omega_i,\Gamma}(t) \underset{\Gamma\gg\gamma}{\approx} E_0^2 \frac{\Gamma^2}{2} \int_0^t dt_1 \int_0^{t_1} dt_2\, e^{-\Gamma(t_1+t_2)/2} \{\cos\left[\Delta_i(t_1-t_2)\right] - \sin[\Delta_i(t_1-t_2)]\Delta\phi(t_1,t_2,t)\} = \langle I_{\omega_i,\Gamma}\rangle + \delta I_{\omega_i,\Gamma}(t) \tag{11}$$

where

$$\langle I_{\omega_i,\Gamma}\rangle = E_0^2 \frac{\Gamma^2}{2} \int_0^t dt_1 \int_0^{t_1} dt_2\, e^{-\Gamma(t_1+t_2)/2} \cos\left[\Delta_i(t_1-t_2)\right] = E_0^2 \frac{(\Gamma/2)^2}{\Delta_i^2 + (\Gamma/2)^2} \tag{12}$$

(in agreement with Eq. (9) in the limit $\Gamma \gg \gamma$) and

$$\delta I_{\omega_i,\Gamma}(t) = -E_0^2 \frac{\Gamma^2}{2} \int_0^t dt_1 \int_0^{t_1} dt_2\, e^{-\Gamma(t_1+t_2)/2} \sin[\Delta_i(t_1-t_2)]\Delta\phi(t_1,t_2,t)\,. \tag{13}$$

Since this equation changes sign when Δ_i changes sign, we observe that, in this limit, the fluctuations around the mean value in opposite sides of the spectrum are perfectly anticorrelated at all times:

$$\delta I_{\omega_0-\omega,\Gamma}(t) = -\delta I_{\omega_0+\omega,\Gamma}(t)\,, \tag{14}$$

and $g^{(2)}_\Gamma(\omega_0+\omega, \omega_0-\omega, \tau=0)$ is lower than one:

$$g^{(2)}_\Gamma(\omega_0+\omega, \omega_0-\omega, \tau=0) = 1 + \frac{\langle \delta I_{\omega_0-\omega,\Gamma} \delta I_{\omega_0+\omega,\Gamma}\rangle}{\langle I_{\omega_0-\omega,\Gamma}\rangle \langle I_{\omega_0+\omega,\Gamma}\rangle} = 1 - \frac{\langle \delta I^2_{\omega_0+\omega,\Gamma}\rangle}{\langle I_{\omega_0+\omega,\Gamma}\rangle^2} < 1\,. \tag{15}$$

II. Frequency correlations of quantum states

The formal theory of time and frequency resolved correlations is well-established since the 80s[2–5]. The two-photon frequency correlations is expressed as (Eq. (2) of the text):

$$g^{(2)}_\Gamma(\omega_1,t_1;\omega_2,t_2) = \frac{\langle : \mathscr{T}\left[\prod_{i=1}^2 \hat{E}_{\omega_i,\Gamma}(t_i) \hat{E}^+_{\omega_i,\Gamma}(t_i)\right] : \rangle}{\prod_{i=1}^2 \langle \hat{E}_{\omega_i,\Gamma}(t_i) \hat{E}^+_{\omega_i,\Gamma}(t_i)\rangle}, \tag{16}$$

where

$$\hat{E}_{\omega_i,\Gamma}(t_i) = \frac{\Gamma}{2} \int_0^\infty e^{-i\omega_i t} e^{-\Gamma t/2} \hat{E}(t_i - t)\, dt \tag{17}$$

is the field of frequency component ω_i and width Γ, at time t_i, and $\mathscr{T}$, (resp. :) refer to time (resp. normal) ordering. This must be contrasted with the conventional second-order correlation function:

$$g^{(2)}_0 = \frac{\sum_{n=0}^\infty n(n-1) \langle n|\rho|n\rangle}{(\sum_{n=0}^\infty n \langle n|\rho|n\rangle)^2}\,, \tag{18}$$

that only requires the density matrix ρ to be computed. On the other hand, for $g^{(2)}(\omega_1, T_1; \omega_2, T_2)$, one needs the time dynamics even to compute zero-delay coincidences with $T_1 = T_2$ since one has to integrate over time t, as seen in Eq. (17). When including the frequency information, one must therefore specify which dynamics is bringing the photons from the state towards

the detectors that will correlate them. The fact that such basic physical processes are required to compute the 2PS shows that it is more fundamental in character than the conventional $g^{(2)}$. This is similar to early descriptions by Eberly and Wódkiewicz[6] of photoluminescence spectra of light beyond the Wiener-Khintchin theorem that presupposes no emission. Here too, the necessity to take into account emission and detection of the photons to define a physical spectrum of light was pointed out.

The most basic process to bring a photon from the quantum state toward a detector is spontaneous emission, followed by free propagation towards the detector that performs the frequency-filtering and correlation. This provides us with the simplest dynamics to which one can subject the time evolution of the quantum state of an harmonic oscillator, as ruled by the master equation for its density matrix:

$$\frac{\partial\rho}{\partial t} = \left[\frac{\gamma_a}{2}\mathscr{L}_a + \frac{\gamma_\phi}{2}\mathscr{L}_{a^\dagger a}\right](\rho)\,. \tag{19}$$

We have also included pure dephasing, for reasons explained in the main text. The equation can be integrated in closed form for $\rho_{n,m} = \langle n|\rho|m\rangle$ which takes the form:

$$\dot\rho_{n,m} = -\frac{1}{2}\left[\gamma_a(n+m) + \gamma_\phi(n-m)^2\right]\rho_{n,m} + \gamma_a\sqrt{(m+1)(n+1)}\rho_{n+1,m+1}\,, \tag{20}$$

and that can be solved by recurrence, yielding:

$$\rho_{n,m}(t) = \sum_{k=0}^{\infty}\rho_{k,m-n+k}(0)\sqrt{\binom{k}{n}\binom{m-n+k}{m}}\left(e^{\gamma_a t}-1\right)^{k-n}e^{-\left[\gamma_a(2k+m-n)+\gamma_\phi(n-m)^2\right]t/2}\,. \tag{21}$$

From $\rho(t)$, one can compute all single-time observables, such as the population:

$$n(t) = \langle a^\dagger a\rangle(t) = n(0)\exp(-\gamma_a t)\,, \tag{22}$$

i.e., simple exponential decay, as expected on physical grounds and despite the complicated form of the general solution. The two-photon correlation:

$$g^{(2)}(t) = \frac{\langle a^\dagger a^\dagger aa\rangle(t)}{\langle a^\dagger a\rangle(t)^2} \tag{23}$$

provides an even simpler and stronger result:

$$g^{(2)}(t) = g^{(2)}(0)\,. \tag{24}$$

The photon-statistics is constant with time. One can also compute the two-times correlation function (Eq. (1) of the main text) through the quantum regression theorem (demonstration not given), and find a similarly constrained result:

$$g^{(2)}(t,\tau) = g^{(2)}(0,0)\,. \tag{25}$$

This implies, for instance, $\lim_{\tau\to\infty} g^{(2)}(t,\tau) \neq 1$ for most of the cases, i.e., photons are always correlated. This is reasonable since any two photons emitted by the system come from the same and only initial state which is let to evolve at precisely $t = 0$. Since we are dealing with many closely related variations of $g^{(2)}$, various notations are in order and for what is the central quantity of this work, the zero-delay (coincidence) second order correlation function, we use:

$$g_0^{(2)} \equiv g^{(2)}(t=0,\tau=0)\,. \tag{26}$$

This quantity is usually found in the literature written as $g^{(2)}(0)$.

We now compute the two-photon correlations from an initial state when including the frequency degree of freedom. To keep the discussion as fundamental and simple as possible, we consider here the time-integrated case that disposes of time altogether:

$$\bar g_\Gamma^{(2)}(\omega_1,\omega_2) = \frac{\iint_0^\infty\langle:\mathscr{T}\left[\prod_{i=1}^2\hat E_{\omega_i,\Gamma}(t_i)\hat E^+_{\omega_i,\Gamma}(t_i)\right]:\rangle dt_1dt_2}{\prod_{i=1}^2\int_0^\infty\langle\hat E_{\omega_i,\Gamma}(t_i)\hat E^+_{\omega_i,\Gamma}(t_i)\rangle dt_i}\,. \tag{27}$$

This is equivalent to letting the detectors gather statistical information from photons detected at any time, hence reconstructing the frequency of the photons with full precision. This quantity is the closest one to what an actual experiment would perform,

although other configurations are possible (they would bring us to an essentially identical discussion and conclusions). Applying Eq. (27) to the case of free propagation only ($\gamma_a = 0$ and $\gamma_\phi = 0$) is pathological because the energy is then exactly determined and frequency correlations become trivial or ill-defined in terms of δ functions. The simplest physically sound dynamics is that of a free field that, at least, decays ($\gamma_a \neq 0$ and $\gamma_\phi = 0$). One can then compute a physical frequency-correlation spectrum, and interpret the photons annihilated by the decay as those detected by the apparatus to register the information required to compute the correlations. In this case, corresponding to spontaneous emission of the state, the result is the same for frequencies as Eq. (25):

$$\bar{g}^{(2)}_\Gamma(\omega_1,\omega_2) = g^{(2)}_0\,, \tag{28}$$

as will be shown in next Section. Including dephasing on top of the radiative decay ($\gamma_a \neq 0$ and $\gamma_\phi \neq 0$) brings us to the result:

$$\bar{g}^{(2)}_\Gamma(\omega_1,\omega_2) = g^{(2)}_0 \mathscr{F}_{\Gamma,\gamma_a,\gamma_\phi}(\omega_1,\omega_2)\,, \tag{29}$$

with $\mathscr{F}_{\Gamma,\gamma_a,\gamma_\phi}(\omega_1,\omega_2)$ a *boson form factor*, which is independent of the quantum state ρ in which the system is prepared, and depends only on the dynamics of emission and detection:

$$\mathscr{F}_{\Gamma,\gamma_a,\gamma_\phi}(\omega_1,\omega_2) = \Re\Big\{\frac{(\gamma^2+4\omega_1^2)(\gamma^2+4\omega_2^2)}{2\gamma^2(\gamma+2i\omega_2)}\Big[\frac{\gamma+2\gamma_a}{(\gamma+2\gamma_a)^2+4\omega_1^2}+\frac{\gamma_a}{\gamma+2\gamma_a+2i\omega_2}$$
$$\times\Big(\frac{\gamma+2\gamma_a-i(\omega_1-\omega_2)}{(\gamma+2\gamma_a-2i\omega_1)(\Gamma+\gamma_a-i(\omega_1-\omega_2))}+\frac{\gamma+2\gamma_a+i(\omega_1+\omega_2)}{(\gamma+2\gamma_a+2i\omega_1)(2\gamma-\Gamma-\gamma_a+i(\omega_1+\omega_2))}\Big)\Big]\Big\}+[1\leftrightarrow 2]\,, \tag{30}$$

with $\gamma = \Gamma+\gamma_a+\gamma_\phi$. The previous result, Eq. (28), follows from $\mathscr{F}_{\Gamma,\gamma_a,\gamma_\phi}(\omega_1,\omega_2)$ being unity when $\gamma_\phi = 0$. This is in agreement with the classical case, as has been discussed in the main text. Interestingly, in the quantum case, even for states such as a Fock state that has no well-defined phase, dephasing is still required to measure nontrivial correlations, showing again how the dynamical aspect predominates over the quantum state itself. This reveals some flow dynamics in Wigner space that does not show in the Wigner function itself. The Fock state can thus be contemplated as a superposition of coherent states with definite phases that are dephasing. The interference between the coherent components of the Fock state would then originate the effect that is revealed through the 2PS.

In general, when the physics goes beyond that of the mere emission from a quantum state ρ, and involves virtual processes, dressing of the states, collective emission, stimulated emission and other types of likewise quantum correlations, the standard Glauber's correlation $g^{(2)}_0$ does not simply factorize from $\bar{g}^{(2)}_\Gamma(\omega_1,\omega_2)$. In such cases, the 2PS offers a complex landscape of correlations with strong and characteristic features[7], that can be taken advantage of for distillation[8], strongly-correlated emission[9] and quantum information processing[10]. This is however experimentally outside the scope of this paper which makes the first step in these exciting directions by confirming Eq. (29).

III. Calculation of the Boson form factor.

We now detail the calculation of $\mathscr{F}_{\Gamma,\gamma_a,\gamma_\phi}(\omega_1,\omega_2)$. Let us assume a quantum system described by a set of operators a, σ, etc., acting in a Hilbert space $\mathscr{H}$. In second quantization, these operators define annihilation operators in the Heisenberg picture. All single-time quantities can be obtained from correlators of the type $\langle a^{\dagger\mu}a^\nu\sigma^{\dagger\eta}\sigma^\theta\ldots\rangle$ with μ, ν, η, θ, etc., integers. Let us call $\mathscr{O}$ the set of operators the averages of which correspond to the correlators required to describe the system, i.e., $\mathscr{O}$ includes all the sought observables as well as operators which couple to them through the equations of motion. In the following, we assume, without loss of generality, that a is the mode of interest, the correlations of which are to be computed in time and frequency during its decaying time dynamics.

We define the time-dependent vector $\mathbf{v}$ as:

$$\mathbf{v} = \begin{pmatrix} 1 \\ \langle a\rangle(t) \\ \langle a^\dagger\rangle(t) \\ \langle a^\dagger a\rangle(t) \\ \vdots \end{pmatrix}\,, \tag{31}$$

composed of the mean values of all relevant operators in $\mathscr{O}$ taken in some order, which will be kept for the remainder of the text as starting with the sequence $\mathscr{O} = \{1, a, a^\dagger, a^\dagger a, \ldots\}$. From the master equation, one can define for $\mathscr{O}$ a matrix M which rules the dynamical evolution of $\mathbf{v}$: $\partial_t\mathbf{v} = M\mathbf{v}$, with solution $\mathbf{v} = e^{Mt}\mathbf{v}_0$ and integrated value $\bar{\mathbf{v}} = \int_0^\infty e^{-\lambda t}\mathbf{v}\,dt = \int_0^\infty e^{-\lambda t}e^{Mt}\mathbf{v}_0\,dt = \frac{-1}{M-\lambda\mathbf{1}}\mathbf{v}_0$.

Note that M is typically singular, i.e., $|M| = 0$, therefore we include an exponential decay $e^{-\lambda t}$ that forces the dynamics to die away so that we can enforce $\lim_{t\to\infty} e^{-\lambda t}\mathbf{v} = 0$. At the end of the calculations we take the limit $\lambda \to 0$.

We now consider two sensors ς_i, $i = 1, 2$, with frequencies ω_i and linewidths Γ_i coupled to the system with strength ε_i such that the dynamics of the system is probed but is otherwise left unperturbed. In that limit, it was shown in Ref.[11] that the frequency correlations of the output field could be mapped exactly to the correlations between the sensors:

$$\bar{g}^{(2)}_{\Gamma}(\omega_1, \omega_2) = \frac{\iint_0^\infty \langle : \mathscr{T}\left[\prod_{i=1}^2 \hat{E}_{\omega_i,\Gamma}(t_i)\hat{E}^+_{\omega_i,\Gamma}(t_i)\right] : \rangle dt_1 dt_2}{\prod_{i=1}^2 \int_0^\infty \langle \hat{E}_{\omega_i,\Gamma}(t_i)\hat{E}^+_{\omega_i,\Gamma}(t_i)\rangle dt_i} = \frac{\iint_0^\infty \langle n_1(t_1) n_2(t_2)\rangle dt_1 dt_2}{\int_0^\infty \langle n_1(t_1)\rangle dt_1 \int_0^\infty \langle n_2(t_2)\rangle dt_2} \tag{32}$$

where $n_i = \varsigma_i^\dagger \varsigma_i$. To compute this quantity, we introduce a sensing vector $\mathbf{w}$ of steady state correlators, by multiplying $\varsigma_1^{\dagger\mu_1}\varsigma_1^{\nu_1}\varsigma_2^{\dagger\mu_2}\varsigma_2^{\nu_2}$ with the operators in $\mathscr{O}$:

$$\mathbf{w}[\mu_1\nu_1, \mu_2\nu_2] = \begin{pmatrix} \langle \varsigma_1^{\dagger\mu_1}\varsigma_1^{\nu_1}\varsigma_2^{\dagger\mu_2}\varsigma_2^{\nu_2}\rangle(t) \\ \langle \varsigma_1^{\dagger\mu_1}\varsigma_1^{\nu_1}\varsigma_2^{\dagger\mu_2}\varsigma_2^{\nu_2} a\rangle(t) \\ \langle \varsigma_1^{\dagger\mu_1}\varsigma_1^{\nu_1}\varsigma_2^{\dagger\mu_2}\varsigma_2^{\nu_2} a^\dagger\rangle(t) \\ \langle \varsigma_1^{\dagger\mu_1}\varsigma_1^{\nu_1}\varsigma_2^{\dagger\mu_2}\varsigma_2^{\nu_2} a^\dagger a\rangle(t) \\ \vdots \end{pmatrix}, \tag{33}$$

where the indices μ_i and ν_i take the values 0 or 1. The integrated quantity is denoted $\overline{\mathbf{w}}[\mu_1\nu_1, \mu_2\nu_2] = \int_0^\infty e^{-\lambda t}\mathbf{w}[\mu_1\nu_1, \mu_2\nu_2]dt$. Furthermore, we also introduce the two-time correlator vector as:

$$\mathbf{w}'[\mu_1\nu_1, \mu_2\nu_2] = \begin{pmatrix} \langle (\varsigma_1^{\dagger\mu_1}\varsigma_1^{\nu_1})(t)(\varsigma_2^{\dagger\mu_2}\varsigma_2^{\nu_2})(t+\tau)\rangle \\ \langle (\varsigma_1^{\dagger\mu_1}\varsigma_1^{\nu_1})(t)(\varsigma_2^{\dagger\mu_2}\varsigma_2^{\nu_2} a)(t+\tau)\rangle \\ \langle (\varsigma_1^{\dagger\mu_1}\varsigma_1^{\nu_1})(t)(\varsigma_2^{\dagger\mu_2}\varsigma_2^{\nu_2} a^\dagger)(t+\tau)\rangle \\ \langle (\varsigma_1^{\dagger\mu_1}\varsigma_1^{\nu_1})(t)(\varsigma_2^{\dagger\mu_2}\varsigma_2^{\nu_2} a^\dagger a)(t+\tau)\rangle \\ \vdots \end{pmatrix}, \tag{34}$$

with a two-time integral as $\overline{\overline{\mathbf{w}}}[\mu_1\nu_1, \mu_2\nu_2] = \iint_0^\infty e^{-\lambda t}e^{-\lambda\tau}\mathbf{w}'[\mu_1\nu_1, \mu_2\nu_2]dt d\tau$. Finally, we define two matrices, $T_\pm$, which, when acting on $\mathbf{v}$ or $\mathbf{w}$, introduce an extra $a^\dagger$ for T_+ and an a for T_-, keeping normal ordering. These matrices always exist, in infinite or in truncated Hilbert spaces (where, if truncation is to order n, a^n is an operator in $\mathscr{O}$ but $a^{n+1} = 0$).

In the regime under consideration, the population $\langle \varsigma_i^\dagger \varsigma_i\rangle \ll 1$ and the equations of motion are valid to leading order in $\varepsilon_{1,2}$:

$$\begin{aligned} \partial_t \mathbf{w}[\mu_1\nu_1, \mu_2\nu_2] = \{M + [(\mu_1 - \nu_1)i\omega_1 - (\mu_1 + \nu_1)\frac{\Gamma_1}{2} + (\mu_2 - \nu_2)i\omega_2 - (\mu_2 + \nu_2)\frac{\Gamma_2}{2}]\mathbf{1}\}\mathbf{w}[\mu_1\nu_1, \mu_2\nu_2] \\ + \mu_1(i\varepsilon_1 T_+)\mathbf{w}[0\nu_1, \mu_2\nu_2] + \nu_1(-i\varepsilon_1 T_-)\mathbf{w}[\mu_1 0, \mu_2\nu_2] + \mu_2(i\varepsilon_2 T_+)\mathbf{w}[\mu_1\nu_1, 0\nu_2] + \nu_2(-i\varepsilon_2 T_-)\mathbf{w}[\mu_1\nu_1, \mu_2 0]. \end{aligned} \tag{35}$$

Since the sensors have exactly zero population at $t = 0$ and at $t = \infty$, i.e., $\overline{w}(0) = \overline{w}(\infty) = 0$, we easily obtain the solution for $\overline{\mathbf{w}}$ by formal integration of these equations, noting that $\int_0^\infty e^{-\lambda t}\partial_t \mathbf{w}\, dt = \lambda\overline{\mathbf{w}}$:

$$\begin{aligned} \overline{\mathbf{w}}[\mu_1\nu_1, \mu_2\nu_2] = \frac{-1}{M + [(\mu_1 - \nu_1)i\omega_1 - (\mu_1 + \nu_1)\frac{\Gamma_1}{2} + (\mu_2 - \nu_2)i\omega_2 - (\mu_2 + \nu_2)\frac{\Gamma_2}{2} - \lambda]\mathbf{1}} \\ \times \Big\{\mu_1(i\varepsilon_1 T_+)\overline{\mathbf{w}}[0\nu_1, \mu_2\nu_2] + \nu_1(-i\varepsilon_1 T_-)\overline{\mathbf{w}}[\mu_1 0, \mu_2\nu_2] + \mu_2(i\varepsilon_2 T_+)\overline{\mathbf{w}}[\mu_1\nu_1, 0\nu_2] + \nu_2(-i\varepsilon_2 T_-)\overline{\mathbf{w}}[\mu_1\nu_1, \mu_2 0]\Big\}. \end{aligned} \tag{36}$$

This produces exactly the same type or recursive relation between the integrated correlators that one has in the case of a steady state (under continuous pumping)[11]. The only difference appears when reaching the last vector in the recursive chain, that now is given by $\overline{\mathbf{w}}[00, 00] = \overline{\mathbf{v}}$ instead of the steady state value.

For the two-time correlator, we have:

$$\begin{aligned} \partial_\tau \mathbf{w}'[\mu_1\nu_1, \mu_2\nu_2] = \{M + [(\mu_2 - \nu_2)i\omega_2 - (\mu_2 + \nu_2)\frac{\Gamma_2}{2}]\mathbf{1}\}\mathbf{w}'[\mu_1\nu_1, \mu_2\nu_2] \\ + \mu_2(i\varepsilon_2 T_+)\mathbf{w}'[\mu_1\nu_1, 0\nu_2] + \nu_2(-i\varepsilon_2 T_-)\mathbf{w}'[\mu_1\nu_1, \mu_2 0]. \end{aligned} \tag{37}$$

The formal double integration of these equations, noting that $\iint_0^\infty e^{-\lambda t} e^{-\lambda\tau} \partial_\tau \mathbf{w}'\, dt\, d\tau = -\overline{\mathbf{w}} + \lambda \overline{\overline{\mathbf{w}'}}$, leads to:

$$\overline{\overline{\mathbf{w}'}}[\mu_1\nu_1, \mu_2\nu_2] = \frac{-1}{M + [(\mu_2 - \nu_2) i\omega_2 - (\mu_2+\nu_2)\frac{\Gamma_2}{2} - \lambda]\mathbf{1}} \\ \times \Big\{ \mu_2 (i\varepsilon_2 T_+) \overline{\overline{\mathbf{w}'}}[\mu_1\nu_1, 0\nu_2] + \nu_2(-i\varepsilon_2 T_-)\overline{\overline{\mathbf{w}'}}[\mu_1\nu_1, \mu_2 0] + \overline{\mathbf{w}}[\mu_1\nu_1, \mu_2\nu_2] \Big\}. \quad (38)$$

The final correlator in the recursive chain is given by $\mathbf{w}'[\mu_1\nu_1, 00] = e^{M\tau}\mathbf{w}[\mu_1\nu_1, 00]$ and the integral, therefore, by:

$$\overline{\overline{\mathbf{w}'}}[\mu_1\nu_1, 00] = \frac{-1}{M - \lambda\mathbf{1}} \overline{\mathbf{w}}[\mu_1\nu_1, 00]. \quad (39)$$

At this stage we are ready to obtain the single-photon and two-photon spectra. The Eberly or time-dependent spectrum of emission of a is given by the average population of any one of the two sensors, say, $\langle n_1\rangle = \langle \varsigma_1^\dagger \varsigma_1\rangle(t)$. Its equation of motion reads $\partial_t \langle n_1\rangle = -\Gamma_1\langle n_1\rangle + 2\Re(i\varepsilon_1\langle \varsigma_1 a^\dagger\rangle(t))$, and with the above notations, the total integrated spectrum is therefore given by:

$$\overline{\langle n_1\rangle} = \lim_{\lambda\to 0} \frac{2}{\Gamma_1} \Re\Big[i\varepsilon_1 T_+ \overline{\mathbf{w}}[01, 00] \Big]_1. \quad (40)$$

The subindex in $[\cdot]_1$ means taking the first element of the resulting vector. Using the solution Eq. (36), the correlator of interest for the spectrum reads:

$$\overline{\mathbf{w}}[01, 0, 0] = \frac{-1}{M + [-i\omega_1 - \frac{\Gamma_1}{2} - \lambda]\mathbf{1}} (-i\varepsilon_1 T_-)\overline{\mathbf{v}}. \quad (41)$$

The two-photon spectrum follows similarly. The integral of the intensity correlations between two sensors, $\langle n_1(t_1) n_2(t_2)\rangle = \langle (\varsigma_1^\dagger\varsigma_1)(t_1)(\varsigma_2^\dagger\varsigma_2)(t_2)\rangle$, is given by $\iint_0^\infty \langle n_1(t_1)n_2(t_2)\rangle dt_1 dt_2 = \lim_{\lambda\to 0}\overline{\overline{\langle n_1 n_2\rangle}} + [1\leftrightarrow 2]$ where $\overline{\overline{\langle n_1 n_2\rangle}} = \iint_0^\infty e^{-\lambda t}e^{-\lambda\tau}\langle n_1(t) n_2(t+\tau)\rangle dt d\tau$ and $[1\leftrightarrow 2]$ means to exchange sensor parameters in the previous expression. The correlator of interest, $\langle n_1(t) n_2(t+\tau)\rangle$, with equation of motion:

$$\partial_\tau \langle n_1(t) n_2(t+\tau)\rangle = -\Gamma_2 \langle n_1(t) n_2(t+\tau)\rangle + 2\Re\Big[i\varepsilon_2 \langle n_1(t)(\varsigma_2 a^\dagger)(t+\tau)\rangle \Big], \quad (42)$$

relies on the vectors $\mathbf{w}'[11, \mu_2\nu_2]$. In particular, $\langle n_1(t)(\varsigma_2 a^\dagger)(t+\tau)\rangle$ is the first element of the vector $T_+\mathbf{w}'[11, 01]$. The integrated correlator reads:

$$\overline{\overline{\langle n_1 n_2\rangle}} = \frac{1}{\Gamma_2 + \lambda}\Big\{ \overline{\langle n_1 n_2\rangle} + 2\Re\Big[i\varepsilon_2 T_+ \overline{\overline{\mathbf{w}'}}[11, 01]\Big]_1 \Big\}. \quad (43)$$

The one-time integrated correlator is given by:

$$\overline{\langle n_1 n_2\rangle} = \frac{1}{\Gamma_1 + \Gamma_2 + \lambda} 2\Re\Big[i\varepsilon_2 T_+ \overline{\mathbf{w}}[11, 01]\Big]_1 + [1\leftrightarrow 2], \quad (44)$$

while the solution for $\overline{\overline{\mathbf{w}'}}[11, 01]$ is:

$$\overline{\overline{\mathbf{w}'}}[11, 01] = \frac{-1}{M + (-i\omega_2 - \frac{\Gamma_2}{2} - \lambda)\mathbf{1}} \Big\{ -i\varepsilon_2 T_- \overline{\overline{\mathbf{w}'}}[11, 00] + \overline{\mathbf{w}}[11, 01] \Big\}. \quad (45)$$

All together, using Eq. (39), we get the final expression for the integrated correlations:

$$\iint_0^\infty \langle n_1(t_1) n_2(t_2)\rangle dt_1 dt_2 = \lim_{\lambda\to 0} \frac{2}{\Gamma_2} \Re\Big[(i\varepsilon_2 T_+) \Big\{ \Big[\frac{1}{\Gamma_1}\mathbf{1} + \frac{-1}{M + (-i\omega_2 - \frac{\Gamma_2}{2})\mathbf{1}} \Big] \overline{\mathbf{w}}[11, 01] \\ + \frac{-1}{M + (-i\omega_2 - \frac{\Gamma_2}{2})\mathbf{1}} (-i\varepsilon_2 T_-) \frac{-1}{M - \lambda\mathbf{1}} \overline{\mathbf{w}}[11, 00] \Big\} \Big]_1 + [1\leftrightarrow 2]. \quad (46)$$

The required vectors are given by the solutions:

$$\overline{\mathbf{w}}[11, 01] = \frac{-1}{M + (-i\omega_2 - \Gamma_1 - \frac{\Gamma_2}{2} - \lambda)\mathbf{1}} \Big\{ -i\varepsilon_2 T_- \overline{\mathbf{w}}[11, 00] - i\varepsilon_1 T_- \overline{\mathbf{w}}[10, 01] + i\varepsilon_1 T_+ \overline{\mathbf{w}}[01, 01] \Big\}, \quad (47)$$

with:

$$\overline{\mathbf{w}}[11,00] = \frac{-1}{M+(-\Gamma_1-\lambda)\mathbf{1}}\Big\{i\varepsilon_1 T_+\overline{\mathbf{w}}[01,00] - i\varepsilon_1 T_-\overline{\mathbf{w}}[10,00]\Big\}, \tag{48a}$$

$$\overline{\mathbf{w}}[10,01] = \frac{-1}{M+(i\omega_1-i\omega_2-\frac{\Gamma_1+\Gamma_2}{2}-\lambda)\mathbf{1}}\Big\{-i\varepsilon_2 T_-\overline{\mathbf{w}}[10,00] + i\varepsilon_1 T_+\overline{\mathbf{w}}[00,01]\Big\}, \tag{48b}$$

$$\mathbf{w}[01,01] = \frac{-1}{M+(-i\omega_1-i\omega_2-\frac{\Gamma_1+\Gamma_2}{2}-\lambda)\mathbf{1}}\Big\{-i\varepsilon_1 T_-\mathbf{w}[00,01] - i\varepsilon_2 T_-\mathbf{w}[01,00]\Big\}. \tag{48c}$$

Finally:

$$\overline{\mathbf{w}}[10,00] = \frac{-1}{M+(i\omega_1-\frac{\Gamma_1}{2}-\lambda)\mathbf{1}} i\varepsilon_1 T_+\overline{\mathbf{v}}, \tag{49a}$$

$$\overline{\mathbf{w}}[00,01] = \frac{-1}{M+(-i\omega_2-\frac{\Gamma_2}{2}-\lambda)\mathbf{1}} (-i\varepsilon_2 T_-)\overline{\mathbf{v}}, \tag{49b}$$

$$\overline{\mathbf{w}}[01,00] = \frac{-1}{M+(-i\omega_1-\frac{\Gamma_1}{2}-\lambda)\mathbf{1}} (-i\varepsilon_1 T_-)\overline{\mathbf{v}}. \tag{49c}$$

With this, we get the final formulas to include in Eq. (46):

$$\begin{aligned}
\overline{\mathbf{w}}[11,01] = {} & -i\varepsilon_1^2\varepsilon_2 \frac{1}{M+(-i\omega_2-\Gamma_1-\frac{\Gamma_2}{2}-\lambda)\mathbf{1}} \\
& \times\Big\{T_-\frac{1}{M+(-\Gamma_1-\lambda)\mathbf{1}}\Big(T_+\frac{1}{M+(-i\omega_1-\frac{\Gamma_1}{2}-\lambda)\mathbf{1}}T_- + T_-\frac{1}{M+(i\omega_1-\frac{\Gamma_1}{2}-\lambda)\mathbf{1}}T_+\Big) \\
& + T_-\frac{1}{M+(i\omega_1-i\omega_2-\frac{\Gamma_1+\Gamma_2}{2}-\lambda)\mathbf{1}}\Big(T_-\frac{1}{M+(i\omega_1-\frac{\Gamma_1}{2}-\lambda)\mathbf{1}}T_+ + T_+\frac{1}{M+(-i\omega_2-\frac{\Gamma_2}{2}-\lambda)\mathbf{1}}T_-\Big) \\
& + T_+\frac{1}{M+(-i\omega_1-i\omega_2-\frac{\Gamma_1+\Gamma_2}{2}-\lambda)\mathbf{1}}T_-\Big(\frac{1}{M+(-i\omega_2-\frac{\Gamma_2}{2}-\lambda)\mathbf{1}} + \frac{1}{M+(-i\omega_1-\frac{\Gamma_1}{2}-\lambda)\mathbf{1}}\Big)T_-\Big\}\frac{1}{M-\lambda\mathbf{1}}\mathbf{v}_0,
\end{aligned} \tag{50}$$

and

$$\overline{\mathbf{w}}[11,00] = -\varepsilon_1^2\frac{1}{M+(-\Gamma_1-\lambda)\mathbf{1}}\Big(T_+\frac{1}{M+(-i\omega_1-\frac{\Gamma_1}{2}-\lambda)\mathbf{1}}T_- + T_-\frac{1}{M+(i\omega_1-\frac{\Gamma_1}{2}-\lambda)\mathbf{1}}T_+\Big)\frac{1}{M-\lambda\mathbf{1}}\mathbf{v}_0. \tag{51}$$

All the previous derivation has been kept at a general level, that could be applied to the spontaneous emission of any system. We now apply it to the case of interest for our previous discussion, namely, the case of an harmonic oscillator with decay and pure dephasing, cf. Eq. (19). In this simple case, the vector the vector $\mathbf{v}$ needed to compute correlators up to second order, truncates naturally at $\langle a^\dagger a^\dagger aa\rangle$ with only 9 elements:

$$\mathbf{v} = \begin{pmatrix} 1 \\ \langle a\rangle(t) \\ \langle a^\dagger\rangle(t) \\ \langle a^\dagger a\rangle(t) \\ \langle a^2\rangle(t) \\ \langle a^{\dagger 2}\rangle(t) \\ \langle a^\dagger a^2\rangle(t) \\ \langle a^{\dagger 2} a\rangle(t) \\ \langle a^{\dagger 2} a^2\rangle(t) \end{pmatrix}. \tag{52}$$

The corresponding matrix M reads $M = \mathrm{Diag}(0, -\frac{\gamma_a+\gamma_\phi}{2}, -\frac{\gamma_a+\gamma_\phi}{2}, -\gamma_a, -(\gamma_a+2\gamma_\phi), -(\gamma_a+2\gamma_\phi), -\frac{3\gamma_a+\gamma_\phi}{2}, -\frac{3\gamma_a+\gamma_\phi}{2}, -2\gamma_a)$.

With this, we can easily compute the integrated vector $\bar{\mathbf{v}}$

$$\bar{\mathbf{v}} = \begin{pmatrix} \frac{1}{\lambda} \\ \frac{2}{\gamma_a+\gamma_\phi+\lambda}\langle a\rangle(0) \\ \frac{2}{\gamma_a+\gamma_\phi+\lambda}\langle a^\dagger\rangle(0) \\ \frac{1}{\gamma_a+\lambda}\langle a^\dagger a\rangle(0) \\ \frac{1}{\gamma_a+2\gamma_\phi+\lambda}\langle a^2\rangle(0) \\ \frac{1}{\gamma_a+2\gamma_\phi+\lambda}\langle a^{\dagger 2}\rangle(0) \\ \frac{2}{3\gamma_a+2\gamma_\phi+\lambda}\langle a^\dagger a^2\rangle(0) \\ \frac{2}{3\gamma_a+2\gamma_\phi+\lambda}\langle a^{\dagger 2} a\rangle(0) \\ \frac{1}{2\gamma_a+\lambda}\langle a^{\dagger 2} a^2\rangle(0) \end{pmatrix}, \tag{53}$$

and apply the procedure that has been previously detailed to arrive at the integrated spectrum of emission:

$$\int_0^\infty \langle n_1(t_1)\rangle dt_1 = \varepsilon^2 \frac{2}{\Gamma\gamma_a}\frac{\gamma/2}{(\gamma/2)^2+\omega_1^2} n_0\,, \tag{54}$$

with n_0 the initial population of the harmonic mode and $\gamma = \Gamma + \gamma_a + \gamma_\phi$ (we also took the coupling to sensors and their decay rates equal for simplicity) and taking the limit $\lambda \to 0$. The integrated correlations can be derived similarly, to provide the more complex expression:

$$\iint_0^\infty \langle n_1(t_1)n_2(t_2)\rangle dt_1 dt_2 = n_0^2 g_0^{(2)} \varepsilon^4 \Re\Big\{ \frac{8}{\Gamma^2\gamma_a^2(\gamma+2i\omega_2)} \Big[\frac{\gamma+2\gamma_a}{(\gamma+2\gamma_a)^2+4\omega_1^2} + \frac{\gamma_a}{\gamma+2\gamma_a+2i\omega_2} \\ \times \Big(\frac{\gamma+2\gamma_a-i(\omega_1-\omega_2)}{(\gamma+2\gamma_a-2i\omega_1)(\Gamma+\gamma_a-i(\omega_1-\omega_2))} + \frac{\gamma+2\gamma_a+i(\omega_1+\omega_2)}{(\gamma+2\gamma_a+2i\omega_1)(2\gamma-\Gamma-\gamma_a+i(\omega_1+\omega_2))} \Big) \Big] \Big\} + [1 \leftrightarrow 2]\,, \tag{55}$$

which, according to Eq. (32), finally provides the analytical expression for the integrated frequency-resolved two-photon spectrum of spontaneous emission of an arbitrary quantum state of the harmonic oscillator with pure dephasing:

$$\bar{g}_\Gamma^{(2)}(\omega_1,\omega_2) = \frac{\iint_0^\infty \langle n_1(t_1)n_2(t_2)\rangle dt_1 dt_2}{\int_0^\infty \langle n_1(t_1)\rangle dt_1 \int_0^\infty \langle n_2(t_2)\rangle dt_2} = g_0^{(2)} \mathscr{F}_{\Gamma,\gamma_a,\gamma_\phi}(\omega_1,\omega_2)\,. \tag{56}$$

The exact expression (30) for $\mathscr{F}_{\Gamma,\gamma_a,\gamma_\phi}(\omega_1,\omega_2)$ follows straightforwardly from Eqs. (54) and (55). This form factor is plotted in Fig. 1c of the main text, and fulfils the following limits: $\lim_{\Gamma\to\infty}\mathscr{F}_{\Gamma,\gamma_a,\gamma_\phi}(\omega_1,\omega_2) = 1$ (we recover the total integrated correlations when opening the window to include all the frequencies) and $\lim_{\gamma_\phi\to 0}\mathscr{F}_{\Gamma,\gamma_a,\gamma_\phi}(\omega_1,\omega_2) = 1$ (without pure dephasing the 2PS lacks any structure in frequency). More notably $\lim_{\Gamma\to 0}\lim_{\gamma_\phi\to\infty}\mathscr{F}_{\Gamma,\gamma_a,\gamma_\phi}(\omega_1,\omega_2) = 1 + \delta_{\omega_1,\omega_2}$, which recovers indistinguishability bunching, the factor 2!, for equal frequencies, and otherwise uncorrelated photons, 1, for different frequencies.

IV. Dynamics of an out-of-equilibrium polariton condensate.

While we have dealt above with the 2PS of spontaneous emission exactly, it is clear that even in this simple case, the exact calculation is an awkward process. For the situation of our experiment, which corresponds instead to a steady state, we recourse to numerical calculations. Importantly, however, the two situations are not extremely different from a physical point of view. The emission in the experiment indeed corresponds to spontaneous emission from a state whose coherence depends on the degree of condensation. The final phenomenology is extremely similar. We describe our system theoretically by the following minimal model which accounts for all the key ingredients of the experiments:

$$\frac{\partial\rho}{\partial t} = \left[\frac{\gamma_a}{2}\mathscr{L}_a + \frac{\gamma_b}{2}\mathscr{L}_b + \frac{P_b}{2}\mathscr{L}_{b^\dagger} + \frac{P_{ba}}{2}\mathscr{L}_{a^\dagger b} \right](\rho)\,, \tag{57}$$

where ρ is the combined reservoir-condensate density matrix defined on the Hilbert space of two bosonic fields, since we describe both the BEC and the exciton reservoir by two harmonic modes a and b, which obey bosonic algebra $[c,c^\dagger] = 1$, with $c = a,b$. In the rotating frame of the frequency of the condensate, the dynamics is purely dissipative. Both modes lose particles, with decay rate γ_c, described by Lindblad terms: $\sum_{c=a,b}\frac{\gamma_c}{2}\mathscr{L}_c(\rho)$, where $\mathscr{L}_c(\rho) = 2c\rho c^\dagger - c^\dagger c\rho - \rho c^\dagger c$. The excitation is

through the incoherent injection of reservoir excitons at a rate P_b with the accompanying Lindblad term $\frac{P_b}{2}\mathscr{L}_{b^\dagger}(\rho)$. The transfer of particles from the reservoir to the condensate, typically assumed to be phonon mediated, is described by the incoherent relaxation mechanism from a to b, described by a crossed Lindblad term $(P_{ba}/2)\mathscr{L}_{a^\dagger b}(\rho)$[12]. In an open system, such a reduced system is enough to capture the physics of condensation that otherwise requires a macroscopic reservoir with N states and $N\to\infty$ to achieve coherence buildup[13]. This model has the minimum, but also all, ingredients to explain the core physical processes that take place within our experimental conditions. It accounts successfully for, e.g., line narrowing and transition to lasing/condensation of the mode a when the pumping P_b is high enough, to all orders of the condensate field correlators $N_{ab}[n,0]$, where:

$$N_{ab}[n,m] = \langle (a^\dagger)^n a^n (b^\dagger)^m b^m \rangle\,, \tag{58}$$

with $n,m \in \mathbf{N}$ form a closed set under the dynamics of Eq. (57)[14]. It is therefore also a sound model to compute theoretically the frequency-resolved correlations.

The zero-time delay dynamics is easily obtained:

$$\dot{N}_{ab}[n,m] = -\Big[n\gamma_a + m\big(\gamma_b - P_b + P_{ba}\big) + nmP_{ba}\Big]N_{ab}[n,m] \\ + n^2 P_{ba} N_{ab}[n-1,m+1] + nP_{ba}N_{ab}[n,m+1] + P_b m^2 N_{ab}[n,m-1] - mP_{ba}N_{ab}[n+1,m]\,. \tag{59}$$

Integrating these equations, it is possible to calculate, e.g., the condensate population, $n_a = N_{ab}[1,0]$, the unnormalized second order correlation function at zero delay $G^{(2)}(\tau=0) = N_{ab}[2,0]$ or any other single time correlator. In particular, the steady state is obtained by setting $\dot{N}_{ab}[n,m]=0$ and solving the system of linear equations, which is finite when truncating to a large enough number of excitations. It is well known, and is straightforwardly shown, that $g^{(2)}$ goes from values above 1, when $P_b \ll \gamma_{b,a}$, to 1 when $P_b \gg \gamma_{b,a}$, corresponding to a coherence buildup that accompanies condensation with $n_a \gg 1$ and triggering a dynamics of relaxation dominated by stimulated emission[13].

By following a similar procedure as in the previous section but for the case of a steady state[11] and the Liouvillian of Eq. (57) we can compute (now numerically) the 2PS in this case. Parameters are $\gamma_a = \gamma_b$ throughout, $P_b = 2\gamma_b$, and $P_{ba} = 10\gamma_a$. The filter linewidth is $\Gamma = \gamma_a/2$ throughout except in Fig. 4, where the values used are indicated. The result is shown in Fig. 2b and Fig. 4 of the main text and is indeed qualitatively very similar to the spontaneous emission case of a coherent state (where $g^{(2)}(0)=1$). In fact, the density plots may appear the same, but one can check by a more careful analysis that they are not exactly identical. The physics, however, has the same interpretation: a state with a given $g^{(2)}(0)$ emits photons which, if correlated in frequencies, exhibit an overall bunching when overlapping in time and frequencies, and antibunching when overlapping in time but distinguished in frequencies.

References

1. Neelen, R. C., Boersma, D. M., van Exter, M. P., Nienhuis, G. & Woerdman, J. P. Spectral filtering within the Schawlow-Townes linewidth of a semiconductor laser. *Phys. Rev. Lett.* **69**, 593 (1992).
2. Cohen-Tannoudji, C. & Reynaud, S. Atoms in strong light-fields: Photon antibunching in single atom fluorescence. *Phil. Trans. R. Soc. Lond. A* **293**, 223 (1979).
3. Dalibard, J. & Reynaud, S. Correlation signals in resonance fluorescence : interpretation via photon scattering amplitudes. *J. Phys. France* **44**, 1337 (1983).
4. Knöll, L. & Weber, G. Theory of n-fold time-resolved correlation spectroscopy and its application to resonance fluorescence radiation. *J. Phys. B.: At. Mol. Phys.* **19**, 2817 (1986).
5. Nienhuis, G. Spectral correlations in resonance fluorescence. *Phys. Rev. A* **47**, 510 (1993).
6. Eberly, J. & Wódkiewicz, K. The time-dependent physical spectrum of light. *J. Opt. Soc. Am.* **67**, 1252 (1977).
7. Gonzalez-Tudela, A., Laussy, F. P., Tejedor, C., Hartmann, M. J. & del Valle, E. Two-photon spectra of quantum emitters. *New J. Phys.* **15**, 033036 (2013).
8. del Valle, E. Distilling one, two and entangled pairs of photons from a quantum dot with cavity QED effects and spectral filtering. *New J. Phys.* **15**, 025019 (2013).
9. Sanchez Muñoz, C. *et al.* Emitters of N-photon bundles. *Nat. Photon.* **8**, 550 (2014).
10. Sanchez Muñoz, C., del Valle, E., Tejedor, C. & Laussy, F. Violation of classical inequalities by photon frequency filtering. *Phys. Rev. A* **90**, 052111 (2014).

11. del Valle, E., Gonzalez-Tudela, A., Laussy, F. P., Tejedor, C. & Hartmann, M. J. Theory of frequency-filtered and time-resolved n-photon correlations. *Phys. Rev. Lett.* **109**, 183601 (2012).

12. Holland, M., Burnett, K., Gardiner, C., Cirac, J. I. & Zoller, P. Theory of an atom laser. *Phys. Rev. A* **54**, R1757 (1996).

13. Laussy, F. *Exciton-polaritons in microcavities*, vol. 172, chap. 1. Quantum Dynamics of Polariton Condensates, 1–42 (Springer Berlin Heidelberg, 2012).

14. del Valle, E. *et al.* Dynamics of the formation and decay of coherence in a polariton condensate. *Phys. Rev. Lett.* **103**, 096404 (2009).
